# Continent-wide survey reveals massive decline in African savannah elephants

**DOI:** 10.7717/peerj.2354

**Published:** 2016-08-31

**Authors:** Michael J. Chase, Scott Schlossberg, Curtice R. Griffin, Philippe J.C. Bouché, Sintayehu W. Djene, Paul W. Elkan, Sam Ferreira, Falk Grossman, Edward Mtarima Kohi, Kelly Landen, Patrick Omondi, Alexis Peltier, S.A. Jeanetta Selier, Robert Sutcliffe

**Affiliations:** 1 Elephants Without Borders, Kasane, Botswana; 2 Department of Environmental Conservation, University of Massachusetts Amherst, Amherst, MA, United States; 3 Department of Biosystems Engineering, Forest Resource Management, Gembloux Agro-Bio Tech, University of Liège, Gembloux, Belgium; 4 College of Agriculture and Environmental Sciences, Haramaya University, Dire Dawa, Ethiopia; 5 Africa Program, Wildlife Conservation Society, Bronx, NY, USA; 6 Scientific Services, South African National Parks, Skukuza, South Africa; 7 Faculty of Geo-Information Science and Earth Observation, University of Twente, Enschede, The Netherlands; 8 Mahale-Gombe Wildlife Research Centre, Tanzania Wildlife Research Institute, Kigoma, Tanzania; 9 Division of Species Conservation & Management, Kenya Wildlife Service, Nairobi, Kenya; 10 Air Adventures (Africa) Ltd, Nairobi, Kenya; 11 Division of Biodiversity Monitoring and Assessment, South African National Biodiversity Institute, Pretoria, Gauteng, South Africa; 12 Amarula Elephant Research Programme, School of Life Sciences, University of Kwa-Zulu-Natal, Durban, South Africa

**Keywords:** African elephant, Conservation, Aerial survey, Population decline, Population trend, Carcass ratio, Protected areas, *Loxodonta africana*

## Abstract

African elephants (*Loxodonta africana*) are imperiled by poaching and habitat loss. Despite global attention to the plight of elephants, their population sizes and trends are uncertain or unknown over much of Africa. To conserve this iconic species, conservationists need timely, accurate data on elephant populations. Here, we report the results of the Great Elephant Census (GEC), the first continent-wide, standardized survey of African savannah elephants. We also provide the first quantitative model of elephant population trends across Africa. We estimated a population of 352,271 savannah elephants on study sites in 18 countries, representing approximately 93% of all savannah elephants in those countries. Elephant populations in survey areas with historical data decreased by an estimated 144,000 from 2007 to 2014, and populations are currently shrinking by 8% per year continent-wide, primarily due to poaching. Though 84% of elephants occurred in protected areas, many protected areas had carcass ratios that indicated high levels of elephant mortality. Results of the GEC show the necessity of action to end the African elephants’ downward trajectory by preventing poaching and protecting habitat.

## Introduction

African elephants (*Loxodonta africana*) play important roles in both the natural and human worlds: ecologically as a keystone species (Bond, 1994), economically as drivers of tourism (Brown Jr, 1993), and culturally as icons of the African continent (Carruthers, 2010). Increasingly, however, elephant populations are threatened by poaching for ivory, human-elephant conflict, habitat loss and fragmentation, and isolation of populations (UNEP et al., 2013). Africa may have held over 20 million elephants before European colonization and 1 million as recently as the 1970s (Douglas-Hamilton, 1987; Milner-Gulland & Beddington, 1993). A wave of poaching in the 1970s and 1980s decimated populations in many areas (Douglas-Hamilton, 1987), and a renewed poaching outbreak beginning around 2005 has led to the deaths of an estimated 30,000 elephants per year recently (UNEP et al., 2013; Wittemyer et al., 2014). Despite worldwide attention to the plight of elephants, our knowledge of elephant populations is still fragmentary. Estimates of continental elephant populations range from ∼400,000 to over 630,000 with little consensus as to the actual numbers (IUCN, 2013), and estimates of poaching losses are based on models and carcass counts rather than changes in numbers of live elephants (UNEP et al., 2013; Wittemyer et al., 2014).

A better understanding of the sizes of and trends in elephant populations is critical for setting conservation priorities. African elephants still occupy a vast range, estimated at 2.3–3.4 million km^2^, but many populations are little studied (IUCN, 2013). Each nation within the elephant’s range is responsible for counting its own elephants, so survey frequency, coverage, and quality vary greatly by country. This piecemeal effort hinders elephant conservation, as the data necessary to set priorities for management at a continental scale are out of date for many areas. Likewise, the lack of synchronized counts across countries makes assessing regional or continental trends difficult.

To meet the need for better information about elephant populations, the Great Elephant Census (GEC) was initiated in 2014 to provide a coordinated, timely, high-quality survey of savannah elephants across the African continent. GEC surveys were conducted in 18 countries in 2014–2015, providing one of the best-quality wildlife surveys to date in Africa. Goals of the GEC were (1) to accurately determine the number and distribution of African savannah elephants over the great majority of their range and (2) to provide a baseline on a continental scale for future surveys. The goal of the GEC was not to census all savannah elephants; doing so would have been impractical and prohibitively expensive. Rather, within each country, we focused on the largest and densest populations, with the goal of counting at least 90% of savannah elephants continent-wide. Because the largest elephant populations should have the highest genetic diversity (Frankham, 1996) and are most likely to persist for the long term (Armbruster & Lande, 1993), these populations are the ones most critical for the future survival of elephants.

We also took advantage of the unprecedented scope of the GEC dataset to address two important questions. First, we assessed the effectiveness of protected areas at a continental scale by comparing elephant populations in protected and unprotected areas; previous studies of how protected areas benefit savannah elephants have generally been limited to smaller scales (e.g., Stoner et al., 2007; Ihwagi et al., 2015). Second, we used counts of elephant carcasses and assessment of historical trends to determine how savannah elephant populations have changed across Africa in the past 20 years. Simply counting elephants is not sufficient to assess conservation priorities because the resulting static picture does not reveal which populations are growing or shrinking. Animal population estimates are far more useful when put in the context of recent history. Also, while data from elephant carcasses suggests that a poaching crisis is occurring across Africa (Wittemyer et al., 2014), data on elephant populations themselves has not been available to corroborate the carcass data at a continental scale. Thus, we combined historical estimates of elephant populations with GEC results to understand elephant population trends by country and for the continent.

## Methods

### Study areas and species

Systematists divide African elephants into two distinct types, savannah and forest elephants, which are distinguished by morphology, genetics, and habitat and may be separate species (Roca et al., 2001; Rohland et al., 2010). The GEC focused on savannah elephants because of the relative ease of counting these animals from the air and growing threats over much of their range (Wittemyer et al., 2014). Forest elephants can be surveyed only with labor-intensive ground counts and would have required distinct methods and analyses. Sampling both types of elephants simultaneously was, therefore, not practical.

We originally targeted 21 countries with significant populations of savannah elephants for GEC surveys (Table S1, Fig. S1). This paper includes data from 18 countries. Privately funded surveys were conducted in Namibia, but the Namibian government did not release data to GEC organizers. GEC surveys planned for Central African Republic and South Sudan in 2015 were postponed due to logistical difficulties and armed conflict. We expect surveys in these two countries to be completed in 2016, and when they are complete, results will be available at the GEC website (http://www.greatelephantcensus.com).

Survey areas were selected by a Technical Advisory Team made up of experts in African elephant ecology and conservation. The team prioritized the largest and densest populations of savannah elephants, with the goal of counting at least 90% of savannah elephants continent-wide. Study sites were organized hierarchically. Within countries, areas to be surveyed were divided into ecosystems, generally contiguous areas averaging 19,020 km^2^ (range: 324–105,000 km^2^). Within ecosystems, survey areas were divided into strata averaging 2,081 km^2^. Locations of stratum and ecosystem boundaries were determined by survey teams, as most teams had previous experience conducting surveys in the locations surveyed for the GEC. Strata often corresponded to geographic, ecological, or political boundaries and were generally designed to minimize internal variation in the density of elephants. For most ecosystems, ecosystem and stratum boundaries used in past surveys were used again on the GEC to allow comparison with past results. Likewise, transect orientation and methods (total counts vs. sample counts and transects vs. blocks) generally matched past practices. As an example of GEC survey design, Fig. S2A shows the stratum design and transect locations for the northern Botswana ecosystem. The entire survey area of 98,307 km^2^ was divided into 48 strata averaging 2,048 km^2^ in area. Stratum boundaries generally followed the boundaries of protected areas or wildlife management areas, and most boundaries were unchanged from the 2010 aerial survey of the same region.

Results for each ecosystem were summarized in a survey report written by the survey team (see below). Habitats surveyed included savannah, grassland, marshes, brush, and open forests. Approval to conduct aerial surveys was granted by the relevant governmental authority in each country where surveys were flown.

### Aerial survey methods

GEC surveys were conducted by a partner research organization or government agency in each country, with all survey teams led by experienced surveyors. Surveys were held to consistent standards via guidelines developed by the GEC Technical Advisory Team and based on those developed for the Monitoring the Illegal Killing of Elephants (MIKE) program (Craig, 2012). Adherence to the standards was contractually obligated for partner organizations (with two exceptions; see below). Standards required: (1) use of the latest technology such as GPS receivers, digital cameras to verify herd counts, voice recorders to document observations, and laser altimeters to ensure that flight altitudes were within standards; (2) adherence to specified flight parameters (height, speed, search rate) to reduce the likelihood of observers’ missing elephants during surveys; (3) sound survey design, including appropriate stratification and full coverage of study areas; (4) use of experienced, well-trained crews as well as survey schedules that minimized crew fatigue; and (5) appropriate analytic methods for estimating elephant populations and carcass ratios. Full standards are available in Article S1.

South Africa was a limited participant in the GEC. Partners in South Africa provided survey data from the Kruger National Park and Tuli ecosystems, but survey teams were not required to follow all GEC procedures. These areas have a long history of surveys (Whyte, Van Aarde & Pimm, 2003), so we did not require that they alter their methods to match GEC standards.

GEC surveys utilized fixed-wing aircraft in all areas except Kruger National Park. In keeping with past practices, Kruger utilized helicopters to conduct a total count of elephants by searching along drainages; helicopters allow for heightened visibility in the rough terrain and dense vegetation of the park. On GEC sites, each stratum was surveyed with one of two survey methods: total count, a complete census of all elephants present using closely spaced transects, or sample count, counting elephants on a subset of a stratum and then extrapolating to the entire stratum. For GEC surveys, sample counts typically covered 5 to 20 percent of a stratum. On sample counts, survey intensity, the percentage of the stratum actually sampled, generally increased with the expected number of elephants in a stratum, as greater effort in larger populations reduces the variance in ecosystem population estimates (Norton-Griffiths, 1978). As an example, for the northern Botswana survey, survey intensity was highest in the two regions where elephant populations were expected to be especially dense, the Okavango Delta in the western part of the ecosystem and the Chobe River region in the northeastern part of the ecosystem (Fig. S2A).

On GEC surveys, sample counts that utilized transects had nominal strip widths of 150–200 m on either side of the plane at the target altitude of 91.4 m. Actual strip widths were estimated by repeated calibration flights at varying altitudes over a runway marked at 10-m intervals. Transects were generally oriented perpendicularly to rivers or ecological gradients to minimize the variation in elephant density between transects (Fig. S2B). In mountainous areas, block counts were used instead of transect counts. During survey flights, observers took photographs of larger herds to ensure that herd sizes were estimated accurately.

In addition to counting live elephants, GEC surveys also counted elephant carcasses (except in five ecosystems where carcasses were not recorded). Dead elephants remain visible for several years after dying, so the “carcass ratio,” the number of dead elephants divided by the sum of live + dead elephants, should be correlated with recent mortality rates. Carcass ratios are often used as an index of population growth rates, as ratios <8% are typical of stable or growing populations over the previous four years, while higher carcass ratios may indicate mortality in excess of births in the previous four years (Douglas-Hamilton & Burrill, 1991). Most survey teams divided carcasses into “fresh” and “old” categories, with fresh carcasses having flesh still attached. Survey teams also counted other large and medium-sized mammal species, including livestock. In some ecosystems, teams counted large birds such as ostrich (*Struthio camelus*) or southern ground-hornbill (*Bucorvus leadbeateri*).

Most surveys were conducted during the local dry season, when clear weather and lack of leaves on deciduous trees make elephants more visible. A few surveys took place during the wet season to allow comparison of GEC results with previous surveys (Table S1). Some surveyed populations straddled international boundaries. In such cases, seasonal movements of elephants could potentially result in the same animals being counted in two different countries. To avoid double-counting elephants in these situations, in most cases, survey teams along international borders coordinated survey timing so that populations in both countries were counted at roughly the same time.

### Data review and processing

To ensure that surveys met GEC standards, surveys were reviewed at two different levels. First, during surveys, team leaders reviewed flight parameters and other data on a daily basis to determine if any changes in methodology were necessary. To ensure that one observer was not undercounting animals, survey teams compared numbers of herds observed between observers on either side of the plane. Only one instance of major disparity between observers was noted, and data from the observer recording fewer elephants was removed from analysis for this survey. Second, expert reviewers evaluated whether or not quantitative standards such as flight speed and survey scheduling were met and evaluated the overall quality of the survey, including both data and conclusions in the survey team’s report. In a few cases, data that did not meet standards were removed or altered. For instance, in the Laikipia-Samburu ecosystem in Kenya, altitude data were reanalyzed because the laser altimeter malfunctioned during surveys. As discussed above, surveys in South Africa did not receive an outside review. Raw data from each survey were collected in a central repository and will be made available to interested parties on request.

### Population estimates

Because accurately estimating the size of large herds from a moving airplane is difficult, survey teams used photographs taken during flights to correct herd-size estimates made from the aircraft (Norton-Griffiths, 1978). Corrected numbers of elephants sighted were then used to estimate elephant populations. For total counts, survey teams plotted observation locations to remove herds that were potentially double-counted on two transects or by two aircraft (Norton-Griffiths, 1978).

For sample counts, because transects were generally of unequal length within strata, we used a ratio estimator to estimate elephant density (*d*) as well as elephant population size (*Y*) and its variance (Jolly, 1969). Accordingly, for each stratum:

\begin{eqnarray*}\hat {d}= \frac{\sum _{i}{y}_{i}}{\sum _{i}{a}_{i}} \end{eqnarray*}

\begin{eqnarray*}\hat {Y}=A\hat {d} \end{eqnarray*}

\begin{eqnarray*}\mathrm{var}(\hat {Y})= \frac{N(N-n)}{n} ({s}_{y}^{2}-2\hat {d}{s}_{ay}+{\hat {d}}^{2}{s}_{a}^{2}), \end{eqnarray*}

where *y*_*i*_ is the number of elephants counted on transect *i*, *a*_*i*_ is the area sampled on transect *i*, *A* is the total area of the stratum, *n* is the number of transects, *N* is the total number of transects possible in the stratum $(A/\bar {a})$, ${s}_{y}^{2}$ is the variance in elephants observed by transect, *s*_*ay*_ is the covariance between transect area and elephants observed, and ${s}_{a}^{2}$ is the variance in transect area. For total counts, the stratum estimate was simply the number of elephants observed, and the variance was assumed to be 0.

We also estimated elephant populations for larger areas including ecosystems, countries, and the entire GEC. The population estimate for an aggregated area was simply the sum of stratum estimates, and the variance of the aggregated population estimate was the sum of the stratum variances. We calculated 95% confidence intervals for ecosystem, national, or survey-wide population estimates with the percentiles from a standard normal distribution (Cochran, 1977). For analysis purposes, we treated the elephant population of the W-Arly-Pendjari ecosystem in Burkina Faso, Benin, and Niger as a single country. This was the only ecosystem surveyed in those three countries, and elephants there move freely between the three nations. Thus, the three countries were considered one in our analyses and are referred to as “W. Africa” in country-level results.

We estimated numbers of carcasses in the same way as for elephants, and carcass ratios were calculated as the estimated number of carcasses divided by the estimated number of live elephants plus carcasses. The variance of a carcass ratio was the binomial variance multiplied by the finite population correction for the region. For surveys that distinguished between fresh and old carcasses, we calculated separate carcass ratios for all carcasses and for fresh carcasses alone.

To determine if protected areas are adequately protecting elephants and how elephants are faring in unprotected areas, we compared the status of elephants in the two types of areas. We used information from the survey team reports and the International Union for the Conservation of Nature’s World Database on Protected Areas (http://www.protectedplanet.net) to determine the protected status of each stratum. Using the methods described above, we estimated elephant populations and carcass ratios separately for protected and unprotected areas by ecosystem, by country, and for the entire GEC. For the Serengeti ecosystem in Tanzania, we were unable to obtain carcass counts in protected and unprotected areas, so we did not include this ecosystem in calculations of carcass ratios by protected status.

Statistical comparison of carcass ratios in protected and unprotected areas was complicated by the fact that most ecosystems had lands in both categories. This led to a lack of independence between carcass ratios for the two types of habitats. Ideally, we would have used a paired *t*-test by ecosystem to compare protected and unprotected areas, but this was not possible because some ecosystems were entirely protected or unprotected. Instead, we used a model designed to compare group means for incomplete paired data (Amro & Pauly, 2016). First, we separated the data into ecosystems that had both protected and unprotected areas and ecosystems with only one status. We then calculated standard *t* statistics for carcass ratios for each type of ecosystem. For the ecosystems with protected and unprotected areas, we used a paired *t* statistic to compare the two types of areas. For the ecosystems with only one protected status, we calculated the two-group *t* statistic, comparing protected and unprotected areas. For each *t*-test, we weighted observations by the estimated number of elephants in the ecosystem to prevent small elephant populations from having excessive influence on the results. We then combined the two *t* values to calculate the test statistic, ${T}_{\mathrm{observed}}={t}_{\mathrm{paired}}\sqrt{a}+{t}_{\mathrm{unpaired}}\sqrt{1-a}$, where *a* is a weighting factor equal to 2*n*_paired_∕(*n*_paired_ + *n*), *n* is the total number of carcass ratio estimates in all ecosystems, and *n*_paired_ is the number of carcass ratio estimates for ecosystems with both protected and unprotected areas. To determine the distribution of *T* under a null hypothesis of no difference between protected and unprotected areas, we permuted the protected status of the paired and unpaired data 1,000 times, resulting in 1,000 *T* values. We used the percentile of *T*_observed_ in the 1,000 *T* values to calculate the exact probability of type-I error for *T*_observed_. We repeated this procedure for all carcasses and for fresh carcasses alone.

To determine if GEC surveys met the goal of counting 90% of savannah elephants in survey countries, we used the most recent (2013) population estimates from the African Elephant Database (IUCN, 2013). We summed estimates for ecosystems not surveyed on the GEC within GEC countries and used this number to estimate the proportion of savannah elephants counted on GEC surveys.

### Population trends

To place elephant population estimates from the GEC in context, we estimated population trends for survey areas where historical estimates were available. We obtained these data from GEC survey team reports and, in a few cases, from published literature and unpublished reports (Data S1). Historical data ware available at the stratum level for some ecosystems and at the ecosystem level for others. We used data only from sites with at least two population estimates, including the GEC, since 1995. A 20-year window was appropriate, as earlier estimates may be unreliable because of differences in methodology. Historical data were unavailable for some sites, so trend estimates reflect only sites with data available.

Where possible, we verified that the geographic areas surveyed historically matched GEC study areas by restricting trend estimates to well-defined areas such as national parks or reserves (e.g., Uganda, Tsavo-Amboseli) or by obtaining historical data or reports and directly comparing past and GEC survey areas (e.g., northern Botswana, Laikipia-Samburu, Serengeti, southeast Angola). For some areas, we did not have access to historical data, so we relied on the judgment of survey teams that GEC survey areas were comparable to historical survey areas. Because most GEC surveys were done by survey teams with previous surveying experience in the same ecosystems, we trust that survey teams were familiar with past practices.

To our knowledge, the only ecosystems for which historical and GEC surveys varied noticeably in area were in Tanzania. For this country, trend data were available at the level of the entire ecosystem, and the area surveyed in each ecosystem increased by a mean of 1% per year from 1995–2014 (S Schlossberg, 2016, unpublished data). An obvious solution to the change in area surveyed would be to treat area as an offset in trend analyses. Use of an offset, however, assumes that the density of elephants was equal in surveyed and unsurveyed areas for years prior to the GEC. This assumption is almost certainly untrue, as surveys were generally expanded over time to encompass small, peripheral populations; core elephant populations were surveyed in all years. Consequently, including area as an offset in the analyses for Tanzania would result in an inflated peak population in the early 2000s and an exaggerated decline thereafter. We believe that trend estimates for Tanzania are more accurate without accounting for area surveyed.

We estimated population trends for elephants for 16 different datasets: one for each of the 15 countries with historical data and a continental dataset with all 15 countries combined. To account for sampling error in elephant population estimates, we used a two-stage Monte Carlo process to model trends in each dataset. Model details differed somewhat by country depending on sample size (see below), but all trend models followed the same basic process (see Fig. S3 illustrating the procedure): (1) For each dataset, we generated 1,000 Monte Carlo replicates to account for error in population estimates; (2) We modeled the historical trend for each Monte Carlo replicate, resulting in 1,000 models; (3) To account for error in model predictions, we used a second round of Monte Carlo sampling on the model predictions. This resulted in one predicted series of historical estimates for each of the 1,000 replicates generated in step 2; (4) We made inferences about historical trends based on the distribution of the predicted values from step 3. Simulations showed that this procedure produces nearly unbiased population estimates across the range of sample sizes used in our models (Article S2). For individual countries, predictions for early years of data tended to be imprecise. Thus, estimates of population change from 1995–2014 for individual countries should be viewed cautiously. In the results, we primarily focus on population change in elephants since 2010, as results from this period are both precise and immediately relevant for management of elephant populations.

To generate the Monte Carlo samples for a dataset, for each population estimate, we drew 1,000 random values from a normal distribution with mean equal to the estimate and SD equal to the SE of the estimate. For total counts, the SE was 0, and all Monte Carlo samples were equal to the original estimate. For 8 out of 322 historical data points, we were missing SE estimates for sample counts. To estimate the SE for these data points, we took advantage of the fact that for count data, variance is generally a power function of population size (McArdle, Gaston & Lawton, 1990). Thus, for the 179 sample counts with reported SEs in the full dataset, we regressed the logarithm of variance on the logarithm of the population estimate. This model had *r*^2^ = 0.87. We used the resulting parameters to approximate the SE for the eight historical estimates with missing SE values.

Using a normal distribution for the Monte Carlo samples was appropriate for sample counts because the ratio estimator should have a normal distribution for samples with ≥30 transects (Cochran, 1977). For smaller samples, normal confidence intervals underestimate true intervals by no more than 8% (Stehman & Salzer, 2000). In our trend analysis, small strata had few elephants and, therefore, contributed little to trend estimates (see below). Additionally, correcting confidence intervals for small strata requires access to raw data (Cochran, 1977), which were not available for most historical samples. Thus, we used the normal approximation to the variance for all strata. The Monte Carlo procedure resulted in 1,000 replicate datasets that account for error in the historical and GEC population estimates.

We then modeled trends in elephant populations for each of the 1,000 Monte Carlo replicates. Angola, Cameroon, and the W-Arly-Pendjari ecosystem each had only two data points available, one from the GEC and one historical estimate. For these countries, we simply fit a generalized linear model with Poisson error, essentially connecting the two data points for each country with a log-linear trend curve. For the survey-wide GEC dataset as well as the 12 countries with >2 observations in their datasets, we used generalized additive models (GAMs) to estimate trends. GAMs are semi-parametric models that fit smoothed curves to data, with the degree of smoothness or roughness dependent on a degrees of freedom (*df*) parameter (Wood, 2006). GAMs were appropriate because their shape is determined by the data rather than constrained to a function, and trends for many survey areas were irregular, reflecting poaching outbreaks and elephant recovery at various times over the past 20 years. Like generalized linear models, GAMs can model non-normal distributions of dependent variables and can also incorporate traditional fixed effects that are not smoothed (Wood, 2006). We ran GAMs using the mgcv package (Wood, 2006) in Program R (R Core Team, 2015).

For countries with >1 stratum, GAMs included a fixed effect of stratum/ecosystem. All GAMs included a smoothed year effect based on a thin-plate spline. Because most strata had intermittent surveys ($\bar {x}=5.9$ data points per stratum), this formulation essentially interpolates the missing data for each stratum. For all models except those for Malawi and DR Congo, we used generalized cross-validation (GCV) to determine the *df* for the smooth. For DR Congo and Malawi, GCV was unavailable due to small sample sizes, so we used a fixed *df* = 6. Within most datasets, strata populations varied widely. Consequently, we weighted observations so that populations would contribute to the trend estimate in proportion to their mean size. As a result, trend estimates reflect the entire population being modeled (Link & Sauer, 1998; Jones & Cresswell, 2010). If the mean population estimate across all years with data for stratum *i* was *m*_*i*_, the weight for each observation in stratum *i* was ${m}_{i}/\bar {m}$, where $\bar {m}$ is the mean of *m*_*i*_ across all strata.

For the GAMs, we used Akaike’s Information Criterion (AIC) as well as evaluation of model predictions to choose between Poisson and negative binomial error distributions. Both distributions used a log link function. For each of the 1,000 Monte Carlo replicates for each dataset, we ran the GAM described above twice, once with a negative-binomial error distribution, and once with a Poisson error distribution. We calculated the AIC of each model and then calculated the proportion of the 1,000 Monte Carlo replicates in which the Poisson or negative binomial model had lower AIC. For eight countries plus the entire GEC area, the negative binomial was selected in 100% of replicates, so we used the negative binomial distribution in our final models for these datasets. For Chad, Malawi, and DR Congo, there was some uncertainty, as each distribution had >9% support for each country. For Chad and DR Congo, the Poisson model produced extraordinarily wide confidence intervals on model predictions, with upper limits exceeding 10^6^ elephants for some years, so we used the negative binomial distribution for our final models. For Malawi, results for the two distributions were very similar, and both produced realistic confidence intervals. We elected to use the negative binomial distribution because it produced slightly wider confidence intervals, making it the more conservative choice. After running the final models, checking the fit of all 13,000 GAMs was not possible. Instead, we checked a sample of 10 replicate models from each dataset for heteroscedasticity, normality of residuals on a log scale, overdispersion, and adequacy of the smooth (Fewster et al., 2000; Wood, 2006). We did not detect any substantial departures from model assumptions.

Next, for each of the 1,000 GAM or Poisson regression models for a dataset, we calculated the predicted number of elephants in each stratum and year as well as its standard error. Because most GEC surveys were conducted in 2014, we used 2014 for the end date for predicting population sizes. For each replicate and year, we then summed estimates across strata to generate a predicted population for all areas surveyed in that country (or for the entire GEC). If we had stopped at this point and simply made inferences from the 1,000 resulting time series, we would have ignored error in model predictions. Thus, for each replicate and year, we calculated the variance of the country-wide prediction as the sum of variances for individual strata. For each replicate, we then generated a Monte Carlo sample from the time series, taking a random normal variate with mean equal to the predicted population and SD equal to the SE of the predicted value for each year. The result of this procedure was 1,000 predicted time series that account for both error in the original population estimates and error in model fitting. For each country, we plotted the annual means of the population estimates as well as the empirical 95% confidence intervals.

To determine how quickly elephant populations were changing, we estimated exponential population growth rates (*r*) for each of the 1,000 predicted time series by regressing log-transformed population estimates on year. As above, these estimates should account for error in both historical population estimates and the trend model. Because trends appeared to have changed over time in many regions, we calculated *r* for three different starting points: 1995, 2005, and 2010. For country-level analyses where the first observation in the dataset was later than any of those three years, the lowest starting point used was the first year of observations. We used the mean of the 1,000 *r* values as the estimate for the time series, and we estimated 95% confidence intervals based on the percentiles of the 1,000 replicates. As discussed above, for individual countries, trends beginning in 1995 may be imprecise due to sparseness of data.

As mentioned above, because we did not have access to raw data for most historical population estimates, our analyses assume that the areas surveyed remained more or less constant for each stratum. Consequently, our trend analyses come with the caveat that changes in areas surveyed over time, while unlikely, could have affected our findings and increase the uncertainty in our results. Thus, the trend estimates provided here should be interpreted with the understanding that the historical data could not be examined for consistency.

**Table 1:** GEC study areas and sampling effort by country. Survey intensity is the proportion of the ecosystem sampled on aerial surveys. “W. Africa” refers to the W-Arly-Pendjari Ecosystem in Burkina Faso, Benin, and Niger.

Country	Ecosystem area (km^2^)	Total transect length (km)	Area surveyed (km^2^)	Survey intensity
Angola	43,459	9,443	3,386	8%
Botswana	101,599	31,486	15,458	15%
Cameroon	20,598	4,955	1,509	7%
Chad	9,799	15,967	8,789	90%
DR Congo	9,349	10,209	4,707	50%
Ethiopia	32,791	3,553	32,791	100%
Kenya	85,711	34,505	32,401	38%
Malawi	2,991	1,300	2,991	100%
Mali	3,944	3,096	3,944	100%
Mozambique	101,077	30,749	12,967	13%
South Africa	19,809	18,139	19,809	100%
Tanzania	268,250	74,047	56,902	21%
Uganda	10,938	8,941	4,588	42%
W. Africa	29,981	9,113	2,914	10%
Zambia	84,814	19,065	6,643	8%
Zimbabwe	68,851	19,949	8,441	12%
**TOTAL**	**893,961**	**294,517**	**218,238**	**24%**

**Figure 1:**
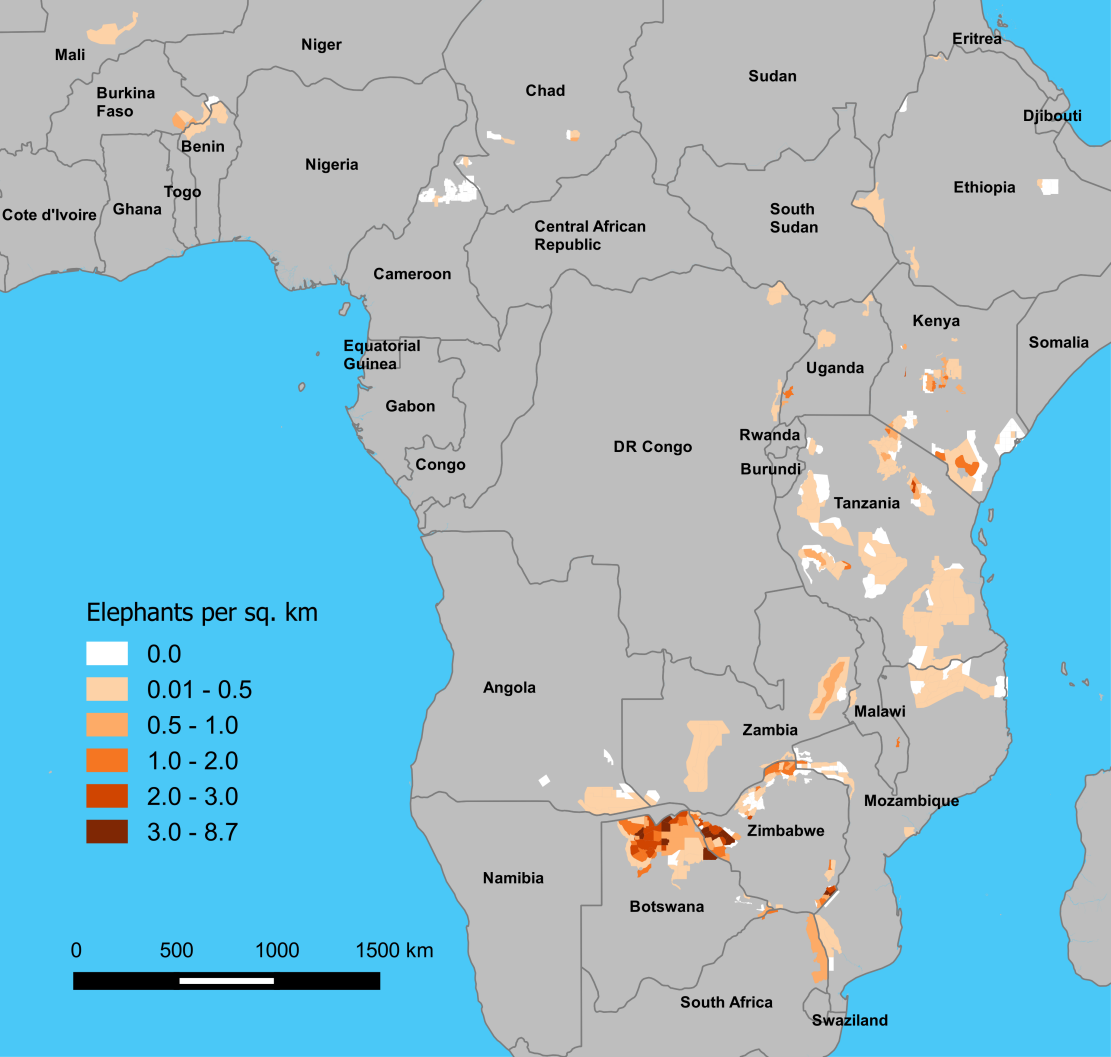
Estimated density of elephants by stratum on GEC survey areas, 2014–2015. Densities are in elephants per km^2^, and estimates are presented by stratum, as defined by survey teams.

## Results

GEC ecosystems totaled 893,961 km^2^ in area (Table 1; Fig. S1, Table S1). Survey teams flew a total of 294,517 km of transects while making 1,573 h of observations, and the area sampled was 218,238 km^2^ or 24% of the total ecosystem area. Elephants occurred in 76% of the survey area, and the total area of strata occupied by elephants was 684,829 km^2^ (Fig. 1). The total population estimate for the GEC was 352,271 ± SE of 9,085 elephants, with 95% confidence interval of 334,464–370,078 elephants (Table 2, Fig. S4, see Table S2 for stratum and ecosystem estimates). Botswana held 37% of this total, with Zimbabwe (23%) and Tanzania (12%) also harboring large populations (Table 2). Estimated density of elephants for the entire GEC was 0.39 elephants km^−2^. Densities were highest in Botswana and Zimbabwe and less than 1 elephant km^−2^ in all other countries (Fig. 1 and Table 2). GEC surveys also recorded 201 poachers’ camps and an estimated 3.39 million head of livestock within GEC study areas.

**Table 2:** Estimated elephant populations and carcass ratios on the GEC, by country.

					All carcasses		Fresh carcasses	
Country	Elephants	SE	95% CI	Density (ele. km^−2^)	Ratio (%)	SE (%)	Ratio (%)	SE (%)
Angola	3,395	797	1,778–5,012	0.08	30.0	2.2	10.4	1.7
Botswana	130,451	6,378	116,957–142,043	1.28	6.9	0.2	0.1	0.02
Cameroon	148	84	12–313	0.01	83.4	4.4	10.3	8.4
Chad	743	0		0.08	17.4	0.0	0.1	0.0
DR Congo	1,959	150	1,773–2,254	0.21	1.4	0.2	0.0	0.0
Ethiopia	799	0		0.02	0.2	0.0	0.1	0.0
Kenya	25,959	1,805	22,421–29,497	0.30	13.0	0.7	0.9	0.2
Malawi	817	0		0.27	2.0	0.0	0.5	0.0
Mali	253	0		0.06	10.0	0.0	0.0	0.0
Mozambique	9,605	1,018	7,610–11,600	0.10	31.6	1.1	3.0	0.5
South Africa	17,433	0		0.88	n/a		n/a	
Tanzania	42,871	3,102	36,792–48,950	0.16	26.4	0.7	1.0	0.2
Uganda	4,864	1,031	2,843–6,885	0.44	0.5	0.2	0.0	0.0
W. Africa	8,911	1,299	6,366–11,457	0.30	9.4	0.1	3.2	0.1
Zambia	21,759	2,310	17,232–26,286	0.26	4.5	0.4	0.1	0.1
Zimbabwe	82,304	4,382	73,715–90,893	1.20	7.8	0.3	0.4	0.1
**TOTAL**	**352,271**	**9,085**	**334,464**–**370,078**	**0.39**	**11.9**	**0.2**	**0.6**	**0.05**

### Carcass ratios

The all-carcass ratio for the entire GEC was 11.9 ± 0.2%. Carcass ratios >8% generally indicate a declining population (Douglas-Hamilton & Burrill, 1991). Ratios varied greatly by country, with the highest ratios in Cameroon (83%), Mozambique (32%), Angola (30%), and Tanzania (26%), suggesting declining populations in these and other countries over the 4 years prior to the GEC (Table 2). The fresh-carcass ratio for the entire GEC was 0.6 ± 0.05% (Table 2). The highest fresh carcass ratios were found in Angola (10%), Cameroon (10%), the W-Arly-Pendjari (WAP) Ecosystem (3%), and Mozambique (3%), suggesting high levels of recent elephant mortality in these countries (Table 2).

### Protected and unprotected areas

Of the total GEC elephant population, an estimated 84% (295,978) were in protected areas while 16% (56,262) were in unprotected areas. Estimated densities were 0.44 elephants km^−2^ in protected areas and 0.23 km^−2^ in unprotected areas. The proportion of elephants in protected areas on GEC sites varied by country, with the majority of elephants in unprotected areas in Mali and Angola and the majority of elephants in protected areas in all other countries (Fig. S5). Survey-wide, carcass ratios were 12.0 ± 0.2% in protected areas and 13.2 ± 0.3% in unprotected areas. This difference was not significant according to a permutation test (*p* = 0.49). Within countries, carcass ratios in protected and unprotected areas did not show any obvious trends (Fig. S6). Survey-wide, fresh-carcass ratios were 0.5 ± 0.05% in protected areas and 1.2 ± 0.2% in unprotected areas. This difference was also not significant (*p* = 0.42).

### Elephant population trends

**Figure 2:**
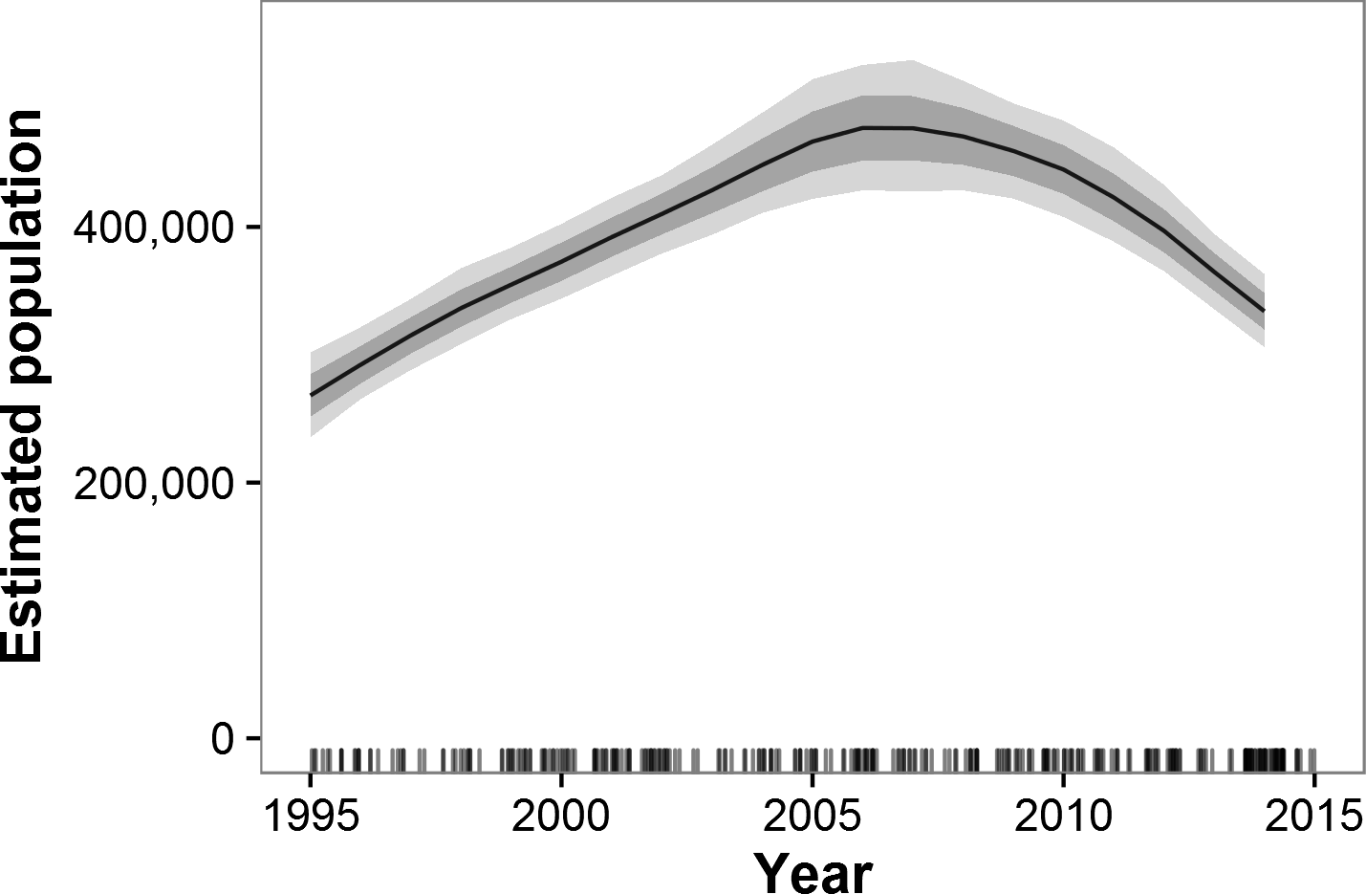
Estimated trends in elephant populations for GEC study areas with historical data available, 1995–2014. Results are based on 1,000 Monte Carlo replicates. Dark shaded area indicates ±1 SD; light shaded area indicates 95% confidence interval. Tick marks on *x*-axis indicate dates of data points used in model; dates are perturbed slightly to prevent overlap.

**Figure 3:**
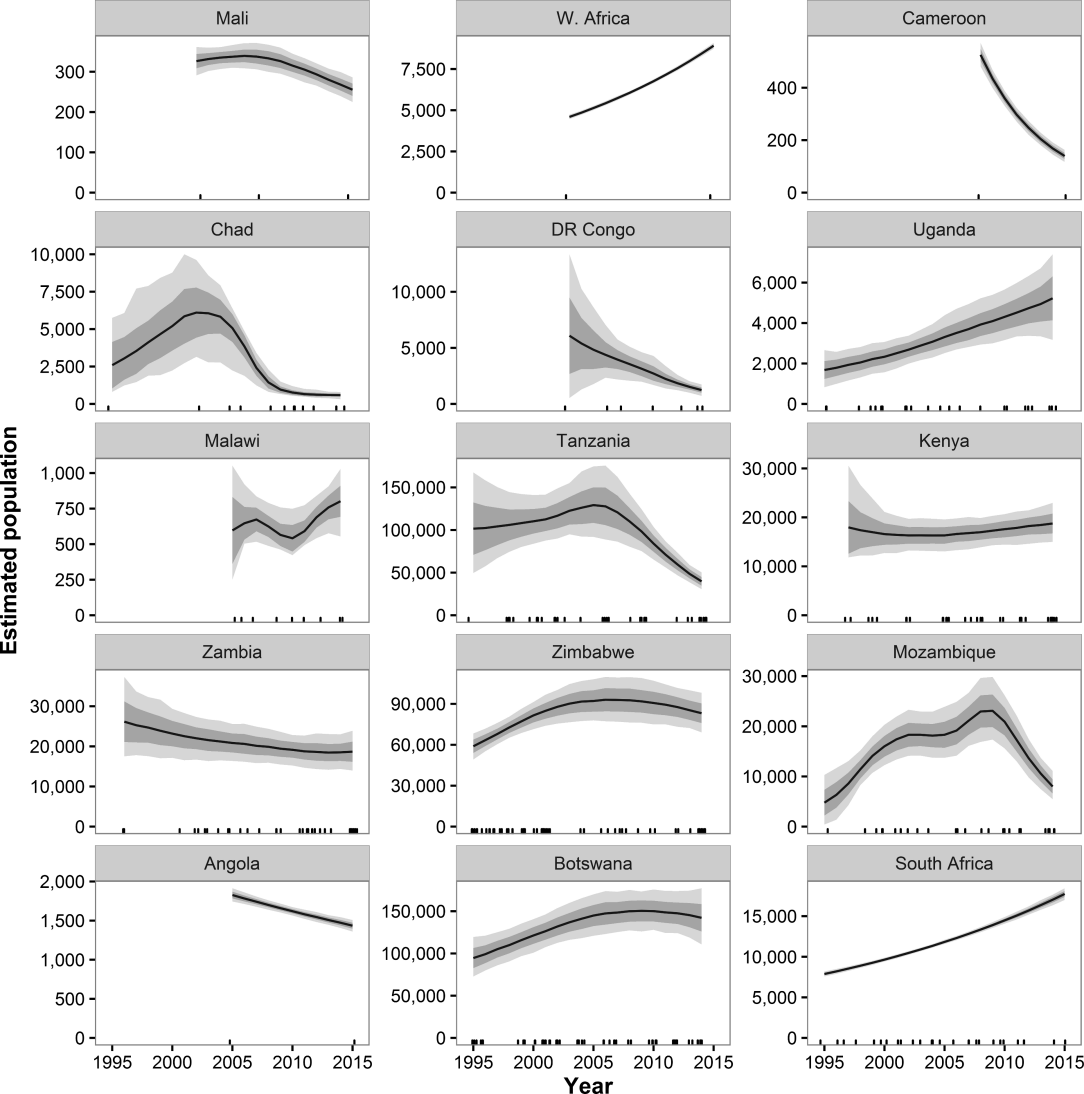
Estimated population trends in GEC study areas by country for sites with historical data available. Results are based on 1,000 Monte Carlo replicates for each country. Dark shaded area indicates ±1 SD; light shaded area indicates 95% confidence interval. Tick marks on *x*-axis indicate dates of data points used in model; dates may be perturbed slightly to prevent overlap. “W. Africa” refers to the WAP ecosystem in Benin, Burkina Faso, and Niger.

Historical data beginning in 1995 and ending with GEC surveys were available for 55 strata in 38 ecosystems in 15 countries (Figs. S7 and S8). These areas represent 87% of the estimated GEC elephant population. Across all GEC countries, our model showed that elephant populations increased from 1995 until approximately 2007 and then began a decline that accelerated after 2010 (Fig. 2). Most individual countries showed similar patterns, with increases prior to roughly 2007 and decreases thereafter (Fig. 3). Exceptions include Malawi, Kenya, Uganda, South Africa, and the WAP Ecosystem, all of which showed increases recently in surveyed areas (Fig. 3). Population growth rates for the entire GEC dataset were slightly above 0 from 1995–2014 (*r* = 0.011, 95% CI [0.006, 0.160]), but populations decreased by an estimated 4% per year (*r* = − 0.044, CI [−0.059, −0.030]) from 2005–2014 and 8% per year (*r* = − 0.078, CI [−0.102, −0.057]) from 2010–2014 (Table S3). According to our trend model, between 2007 and 2014, elephant populations in areas with historical data declined by 144,213 ± SE of 29,253 elephants or 20,602 ± 4,179 elephants per year. Between 2010 and 2014, elephants declined at a rate of 27,691 ± 5,996 elephants per year.

By country, *r* estimates for 2010–2014 were negative in 10 countries and significantly negative in 7 countries; *r* estimates were positive in 4 countries plus the WAP ecosystem and significantly positive in 2 countries plus WAP (Table S3). Countries with significantly declining populations for 2010–2014 had more elephants (58,974) than countries with significantly increasing populations (9,728).

## Discussion

The original goal of the GEC was to count 90% of savannah elephants. Per the African Elephant Database (AED), which compiles elephant population estimates throughout Africa, we estimate that our surveys recorded 93% of savannah elephants in the GEC countries. Within GEC countries, populations that were not surveyed on the GEC were generally small, isolated, and poorly studied (IUCN, 2013). Some of these populations have never been surveyed so that their population sizes are highly speculative. Many other non-GEC populations have not been surveyed in over 10 years. As a result, estimated sizes of many non-GEC populations in the AED are likely too high because effects of recent poaching have not been taken into account. Our estimate that we surveyed 93% of savannah elephants in GEC countries should, therefore, be considered conservative, and the actual percentage is likely higher.

Namibia was the only country with a large savannah elephant population that was not a part of the GEC. Large-scale surveys conducted in Namibia in 2015 produced an estimate of 22,711 elephants (CITES, 2016). When added to the 352,271 elephants estimated on the GEC, this leads to a total estimate of 374,982 savannah elephants. Additional GEC surveys near completion in South Sudan and Central African Republic will increase this total somewhat in the coming months.

### Protected and unprotected areas

Comparing protected and unprotected areas on the GEC demonstrated both the importance of protected areas for the future of savannah elephants and the need to better protect their habitats. A large majority of estimated elephant populations were in protected areas, and in nine countries, all elephants observed occurred in protected habitats (Fig. S5). Undoubtedly, there was some bias towards surveying protected areas in the selection of GEC study sites. Thus, the proportion of all savannah elephants on protected areas is likely overestimated. Assuming that we surveyed 93% of savannah elephants, however, even if all unsurveyed elephant populations are in unprotected areas, the great majority of savannah elephants would still occur in protected areas in GEC countries.

Elephant carcass ratios were not significantly different in protected and unprotected areas. Thus, the high carcass ratios observed on the GEC are not simply due to elephants’ being persecuted outside of protected areas. Rather, carcass ratios of 12–13% suggest that deaths likely exceeded births over the four years preceding GEC surveys in both protected and unprotected areas. For protected areas, the clear implication is that many reserves are failing to adequately shield elephants from poaching and human-elephant conflict (e.g., Gandiwa et al., 2013; Muboko et al., 2014; Booth & Dunham, 2016). We recorded notably high carcass ratios, potentially indicating high poaching levels, in the northern section of Tsavo East National Park, Kenya (52% carcass ratio), Niassa National Reserve, Mozambique (42%), and Rungwa Game Reserve, Tanzania (36%). Heightened anti-poaching measures are needed in these and other protected areas to ensure that they do not become mere “paper parks” for elephants. At the same time, we estimated that over 50,000 savannah elephants occur in unprotected areas. Thus, improved protection for unprotected habitats with substantial numbers of elephants may also benefit the species (De Boer et al., 2013; Ihwagi et al., 2015).

### Trends

Our model of historical trends suggests that from 1995 to approximately 2007, elephant populations were recovering from the poaching outbreak in the 1980s (Douglas-Hamilton, 1987). After that time, however, trends reversed, with large declines observed in many countries and for the GEC survey area as a whole. If populations continue to decline at the 8% rate we estimated for 2010–2014, GEC survey areas will lose half of their savannah elephants every nine years, and extirpation of some populations is possible, especially in countries such as Mali, Chad, and Cameroon with small and isolated savannah elephant populations.

These dramatic declines in elephant populations are almost certainly due to poaching for ivory. Elephant poaching has increased substantially over the past 5–10 years, especially in eastern and western Africa (UNEP et al., 2013; Wittemyer et al., 2014). Wittemyer et al. (2014) estimated that ∼100,000 elephants were poached in Africa between 2010 and 2012. Our trend model indicated a comparable decline of 79,413 elephants on GEC sites with historical data over those three years. Similarly, genetic analysis of intercepted ivory shipments revealed that Mozambique and Tanzania were the primary sources for ivory from savannah elephants (Wasser et al., 2015). According to our trend model, elephant populations in these two countries were declining at 14% and 17% per year, respectively, as of 2014 (Fig. 3). DR Congo, which had the second fastest population decline of any country in our dataset, was also a major origination point for ivory (Wasser et al., 2015). Within Mozambique and Tanzania, the Niassa (Mozambique) and Selous (Tanzania) ecosystems were especially frequent sources of poached ivory, and elephant populations have decreased by over 75% in the past 10 years in these two ecosystems (Fig. S8). Thus, the illegal ivory trade appears to be the major driver of recent population trends in savannah elephants.

These trend estimates come with three important caveats. First, trends for individual strata or ecosystems within countries varied widely. For instance, in Zambia, elephant populations in the West Zambezi ecosystem plummeted from ∼900 in 2004 to 48 in 2015, but populations in the Kafue ecosystem grew by 55% to 6,700 over the same time period. Thus, trends for countries should not be seen as reflecting all constituent ecosystems. Second, not all GEC study areas had historical data available, so trends in some areas are unknown. Still, the declines we observed in Tanzania, Mozambique, DR Congo, and other countries are so large that additional historical data would be very unlikely to change any conclusions at the continental scale. Our trend estimates are further supported by the negative correlation we observed between GEC carcass ratios and elephant population change over the previous four years (Fig. S9). DR Congo and Chad are outliers for this relationship, but survey teams in both countries reported that carcasses were greatly underestimated. Third, we did not have access to raw historical data for most ecosystems. Consequently, we cannot verify that areas surveyed were constant over time. Thus, results of the trend analysis may have some error due to changes in survey area affecting elephant population estimates. Still, we have reason to believe that the most of the historical record we used is reliable. For 67% of the 55 strata used in the historical analysis, either the stratum was a well-defined area such as a national park, or we were able to use historical data to verify that GEC and historical study areas matched.

Poaching is not the only anthropogenic factor affecting elephant populations. The large numbers of livestock observed on our surveys suggest that conflict between elephants and human populations is widespread (UNEP et al., 2013). By 2050, human populations are projected to double in 12 GEC countries (Population Reference Bureau, 2015), and many of these countries already have small elephant populations that may be susceptible to poaching and habitat loss. As human populations grow, so will the potential for human-elephant conflict (Hoare, 1999; Sitati et al., 2003), leading to elephant deaths, loss of habitat to agriculture or fires, and potential extirpation of elephant populations (Hoare & Du Toit, 1999).

### Regional differences in elephant population status

The GEC revealed large regional differences in the status of savannah elephants. Countries in western and central Africa, such as Chad, Cameroon, Mali, and DR Congo, had savannah elephant populations that were small, isolated, and declining in the face of poaching and expanding human populations (Bouché et al., 2011; IUCN, 2013). The WAP population, on the borders of Niger, Burkina Faso, and Benin, is the only savannah elephant population in western or central Africa with over 2,000 elephants. Elephant populations in the WAP ecosystem have grown in recent years, but the high fresh-carcass ratios recorded may be a warning sign of increased poaching that has yet to noticeably affect population estimates.

In eastern Africa, Mozambique and Tanzania have experienced large declines in elephant populations. Though numbers of elephants are still relatively high in these two countries, poaching has had major impacts on elephant populations. Elephant populations elsewhere in eastern Africa, including Kenya, Uganda, and Malawi, show more positive trends recently. Thus, the poaching crisis does not appear to have affected all east African countries equally. In southern Africa, four countries, Botswana, South Africa, Zambia, and Zimbabwe, have relatively large elephant populations and show either increasing trends or mild and non-significant declines recently. According to the Monitoring the Illegal Killing of Elephants program, southern Africa has experienced less poaching than any other part of Africa (CITES, 2014). Angola, however, is an exception, with extremely high carcass ratios and large numbers of fresh carcasses suggesting high levels of ongoing poaching.

## Conclusions

The GEC results come with some caveats. Even with the highest standards, the quality of aerial survey results is limited by the perceptual abilities of human observers, and all observers miss some animals on surveys (Caughley, 1974). A recent study of aerial surveys for elephants found that dense vegetation can cause elephants to go undetected on surveys and that smaller herds are easily missed (S Schlossberg, 2016, unpublished data). As a result, actual elephant populations are likely slightly higher than GEC estimates. Still, by controlling flight speeds and altitudes, we hopefully minimized observer error on surveys. Another caveat of our results is that because we were not able to count forest elephants, found in the rainforests of central Africa, our conclusions apply only to savannah elephants. Forest elephants, however, appear to be highly threatened by habitat loss and poaching, and there is great uncertainty about their current populations (IUCN, 2013; Maisels et al., 2013). Thus, there is a critical need to conduct a high-quality, multi-country census of forest elephants similar to the GEC or the 2003–2005 MIKE survey of the Congo Basin (Blake et al., 2007).

The GEC was the first-ever continental-scale survey of African elephants. These results will serve as a baseline for assessing change in savannah elephant populations throughout Africa. Because elephant populations can change rapidly, surveys on the scale of the GEC should be conducted regularly to measure population trends, gauge the effectiveness of conservation measures, and identify populations at risk of extinction. Future surveys may also allow detection of emerging threats such as drought and climate change. Ideally, results from this survey will encourage people across Africa and around the world to protect and conserve elephant populations. Preliminary results from the GEC have already motivated the governments of Mozambique and Tanzania to implement new measures to stabilize elephant populations (Lopez, 2015; Wildlife Conservation Society, 2015). The future of African savannah elephants ultimately depends on the resolve of governments, conservation organizations, and people to apply the GEC’s findings by fighting poaching, conserving elephant habitats, and mitigating human-elephant conflict. Over 350,000 elephants still roam Africa’s savannahs, but with populations plunging in many areas, action is needed to reverse ongoing declines.

##  Supplemental Information

10.7717/peerj.2354/supp-1 Figure S1 Study ecosystems surveyed on the GEC in 2014–2015 Individual ecosystems are denoted by distinct colors. (A) overview map of the continent. (B), (C), & (D), study ecosystems by region, with names in italics.

10.7717/peerj.2354/supp-2 Figure S2 Northern Botswana example of stratification and transect design as used on the GEC (A) Stratum design and sampling intensity (color) used on the northern Botswana GEC survey in 2014. (B) Transect design used on the northern Botswana GEC survey in 2014. Tracks shown are the actual tracks recorded on survey aircraft.

10.7717/peerj.2354/supp-3 Figure S3 Flow chart illustrating procedures used to estimate trends in elephant populations Hypothetical data (uppermost box) were created for this example, but numbers in boxes in subsequent steps are actual model output using the hypothetical data as input.

10.7717/peerj.2354/supp-4 Figure S4 Estimated elephant population totals by ecosystem on GEC surveys See Fig. S1 for ecosystem names.

10.7717/peerj.2354/supp-5 Figure S5 Estimated elephant populations in protected and unprotected areas by country on GEC surveys Error bars indicate ± 1 SE.

10.7717/peerj.2354/supp-6 Figure S6 Estimated carcass ratios by protected status within countries on GEC surveys (A) ratios for all carcasses. (B) ratios for fresh carcasses. Only countries with elephant populations in both protected and unprotected areas are shown. Error bars indicate ±1 SE. Country codes: AGO–Angola, BWA–Botswana, TCD–Chad, KEN–Kenya, MOZ–Mozambique, TZA–Tanzania, ZWE–Zimbabwe.

10.7717/peerj.2354/supp-7 Figure S7 Study areas used in analysis of historical trends in elephant popualtions, by country Unique colors signify different strata. Strata names are in italics.

10.7717/peerj.2354/supp-8 Figure S8 Historical elephant population estimates used to estimate population trends Data points in 2014–2015 are from the GEC; earlier estimates come from published and unpublished reports and surveys. For Tsavo-Amboseli, the vertical line indicates an additional, non-GEC survey conducted in 2014.

10.7717/peerj.2354/supp-9 Figure S9 Relationship between carcass ratio on GEC surveys and estimated population trends from 2010-2014, by country Country codes: AGO–Angola, BEN–Benin, BWA–Botswana, BFA–Burkina Faso, CAM–Cameroon, TCD–Chad, DRC–DR Congo, KEN–Kenya, MWI–Malawi, MLI–Mali, MOZ–Mozambique, NER–Niger, TZA–Tanzania, UGA–Uganda, ZMB–Zambia, ZWE–Zimbabwe.

10.7717/peerj.2354/supp-10 Table S1 Sampling effort and dates in GEC study areas Abbreviations: R: reserve, NP national park, NR: national reserve, CWA: community wildlife area.

10.7717/peerj.2354/supp-11 Table S2 Elephant population estimates by ecosystem and stratum on GEC study areas

10.7717/peerj.2354/supp-12 Table S3 Estimated exponential population growth rates (*r*) on GEC survey areas by country and start year, with 95% confidence limits Rows in bold indicate *r* significantly >0; rows in italics indicate r significantly <0.

10.7717/peerj.2354/supp-13 Article S1 Great Elephant Census survey standards

10.7717/peerj.2354/supp-14 Article S2 Explanation of simulations used to evaluate Monte Carlo methods discussed in the main text

10.7717/peerj.2354/supp-15 Data S1 GEC survey data Population estimates from GEC survey areas used to calculate country- and continent-wide statistics.

10.7717/peerj.2354/supp-16 Data S2 Historical data used to analyze trends in elephant populations File includes GEC and historical estimates used to estimate trends in elephant populations from 1995-2014.

## Additional Information and Declarations

### Field Study Permissions

The following information was supplied relating to field study approvals (i.e., approving body and any reference numbers): Benin: V/L N°207/15/CENAGREF/DG/RCT/AD 19 May 2015 issued by ANAC Benin Burkina Faso: N°2015-004/ANAC and 2015/005/ANAC (one per aircraft) on the 28 of May 2015 issued by ANAC Burkina Faso Niger: N°00671/ME/SU/DD/SG/DG/EF/DFCAP 21 May 2015 issued by the Ministry in charge of Environment Ethiopia: permit number D29/114/06 Kruger NP, South Africa: surveys conducted under the Mandate of SANParks as the management authority of National Parks. This forms part of normal management and monitoring activities of SANParks. Angola: MoU dated 25 July 2015, signed with the National Director of Biodiversity, under the Ministry of Environment Botswana (northern Botswana): Research Permit Number: EWT8/36/4XXIV, issued from the Ministry of Environment Wildlife and Tourism Kenya (Tsavo-Amboseli and Laikpia-Samburu): MoU signed 24 March 2014, entitled: KWS and EWB collaboration in sample aerial surveys of elephants in open savannah ecosystems in Kenya. Botswana (Tuli): Permission was granted by the Department of Wildlife and National Parks, 18 June 2014 ref no: WP/RES 15/2/2 XXIV (154) South Africa (Tuli): The count was undertaken in collaboration with SANParks, permission through SANParks mandate as with Kruger National Park. Zimbabwe (Tuli): 14 August 2014, Zimbabwe Parks and Wildlife Management Authority, Mr. Alex Dangare and Ms. Kudakwashe Chipunza Mozambique: Permit number 094/MINAG/DNTF/2014, 28-08-2014. And permit number 6077/MINAG/DTNF/2014, 18-09-2014. From Ministerio de Agricultura, Direcção Nacional de Terras e Florestas Zambia: Memorandum of Understanding between Zambia Wildlife Authority, Vulcan Inc., and the Nature Conservancy, dated August 15, 2015. Tanzania: Memorandum of Understanding between Tanzania Wildlife Research Institute and Vulcan Inc. on collaboration in the aerial counting of elephants in Tanzania, dated May 1, 2014. Cameroon: Presidence de la Republique du Cameroon Texte No. 151737/MP/EMP/PR DU 08 AVR 2015 Uganda: May 29, 2014: CAA/UGA/20140529/348E, CAA/UGA/20140529/347E; May 8, 2014: CAA/UGA/20140508/100G, CAA/UGA/20140508/099G Chad: April 1, 2014: permit number 153/MERH/SG/2014 DR Congo (Garamba): as per management contract between ICCN and APN Malawi: Permission to undertake census in Liwonde and Kusungu was granted verbally by Director of DNPW and undertaken by Derek McPherson. For all other countries or ecosystems, to the best of our knowledge, all surveys were flown with full permission from local authorities.

### Data Availability

The following information was supplied regarding data availability: The raw data has been supplied as a Supplemental File.
