# Supplementary figures and images for: Continent-wide survey reveals massive decline in African savannah elephants
Source: PeerJ. 2016 Aug 31;4:e2354. doi: 10.7717/peerj.2354 (PMC5012305; doi:10.7717/peerj.2354)

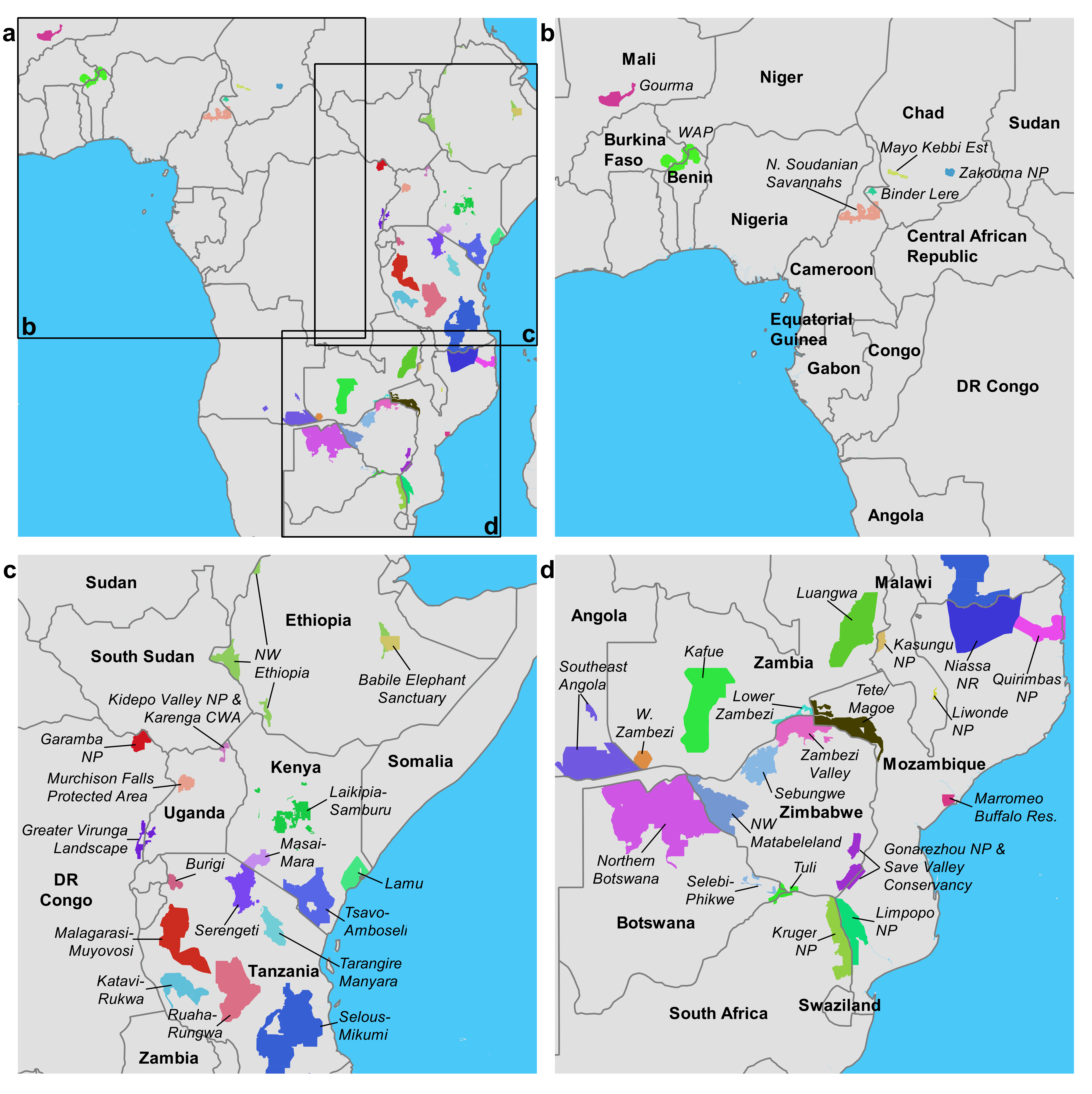

Supplement: Figure S1 — Individual ecosystems are denoted by distinct colors. (A) overview map of the continent. (B), (C), & (D), study ecosystems by region, with names in italics. [file peerj-04-2354-s001.png]

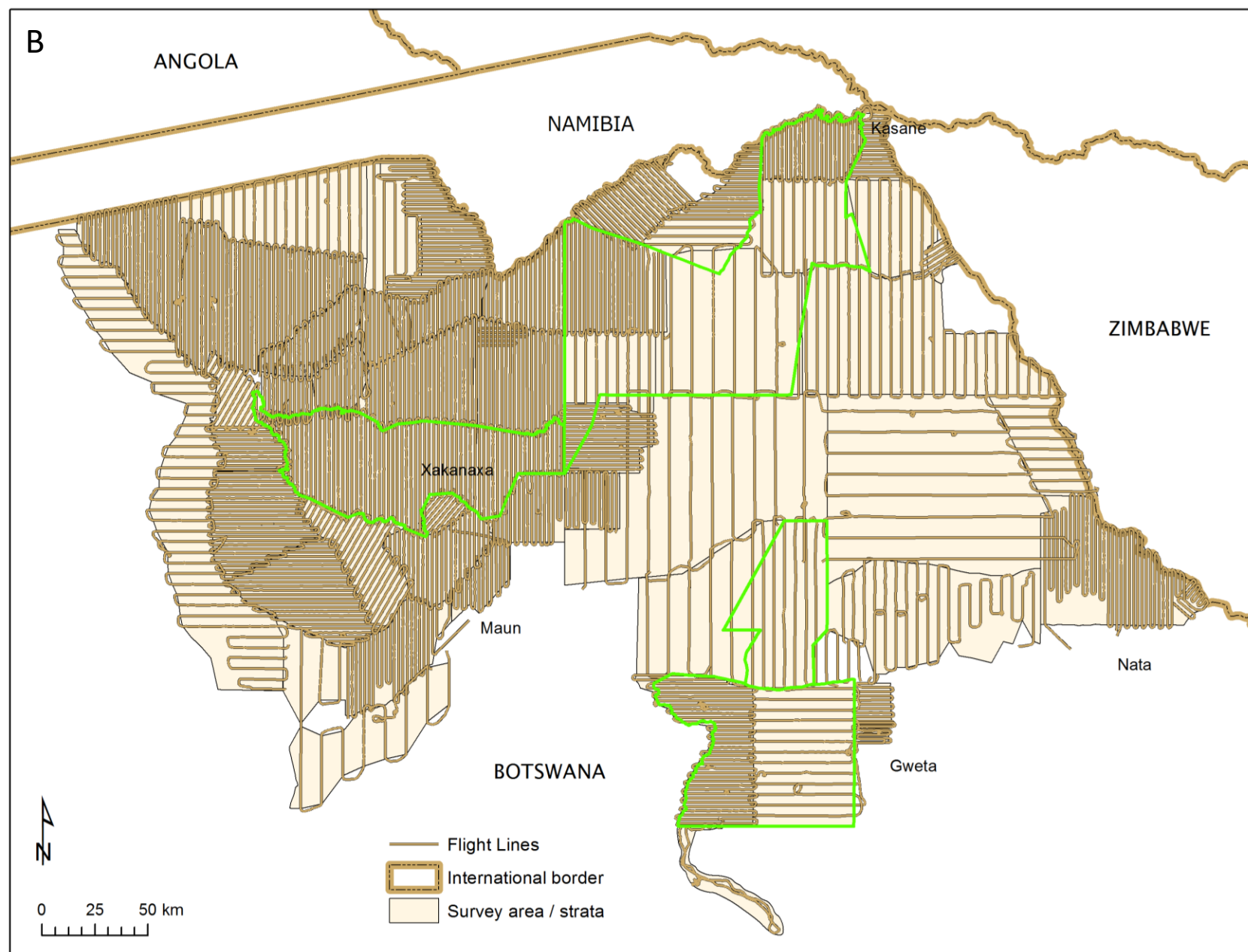

Supplement: Figure S2 — (A) Stratum design and sampling intensity (color) used on the northern Botswana GEC survey in 2014. (B) Transect design used on the northern Botswana GEC survey in 2014. Tracks shown are the actual tracks recorded on survey aircraft. [file peerj-04-2354-s002.pdf]

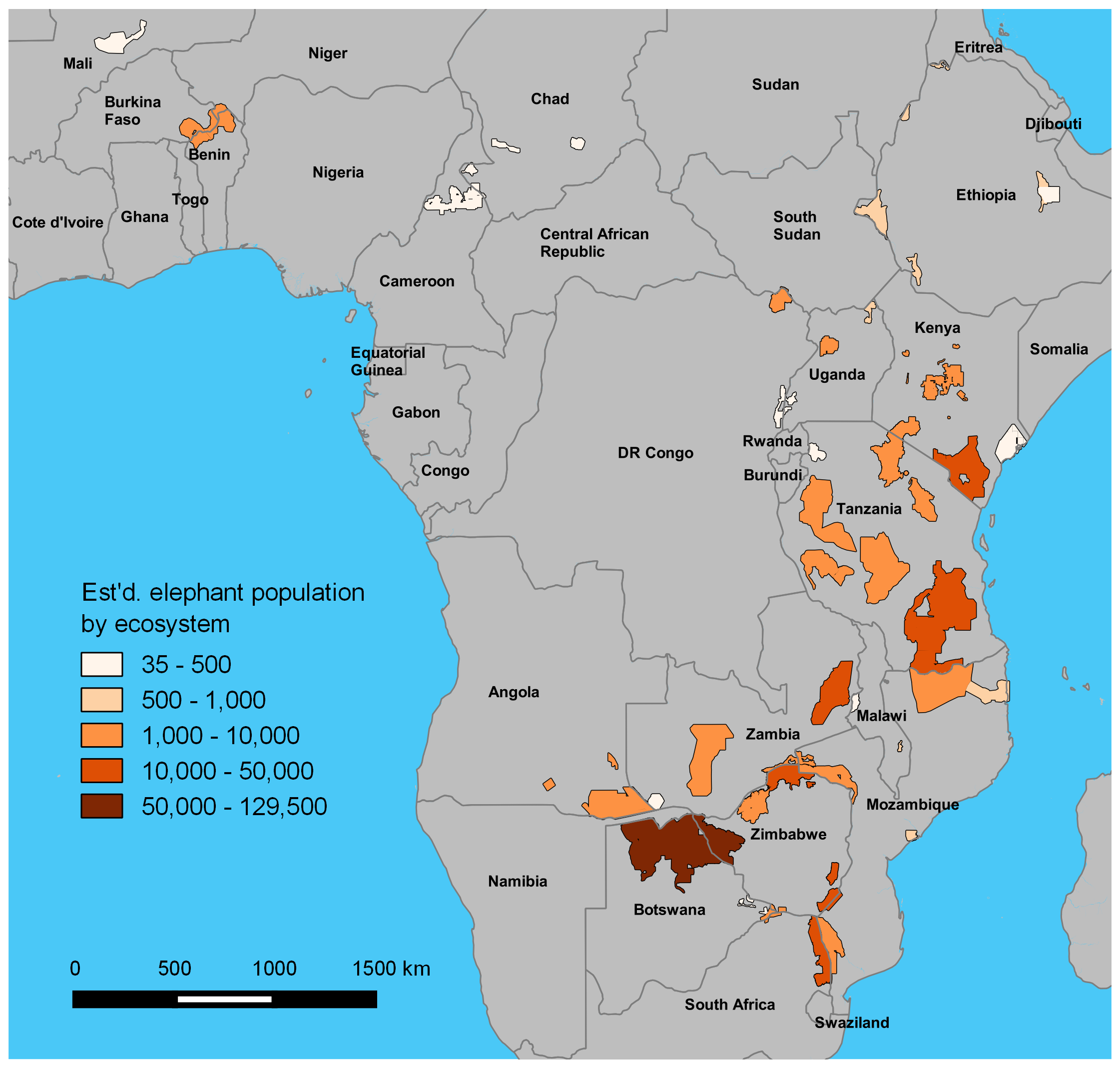

Supplement: Figure S4 — See Fig. S1 for ecosystem names. [file peerj-04-2354-s004.png]

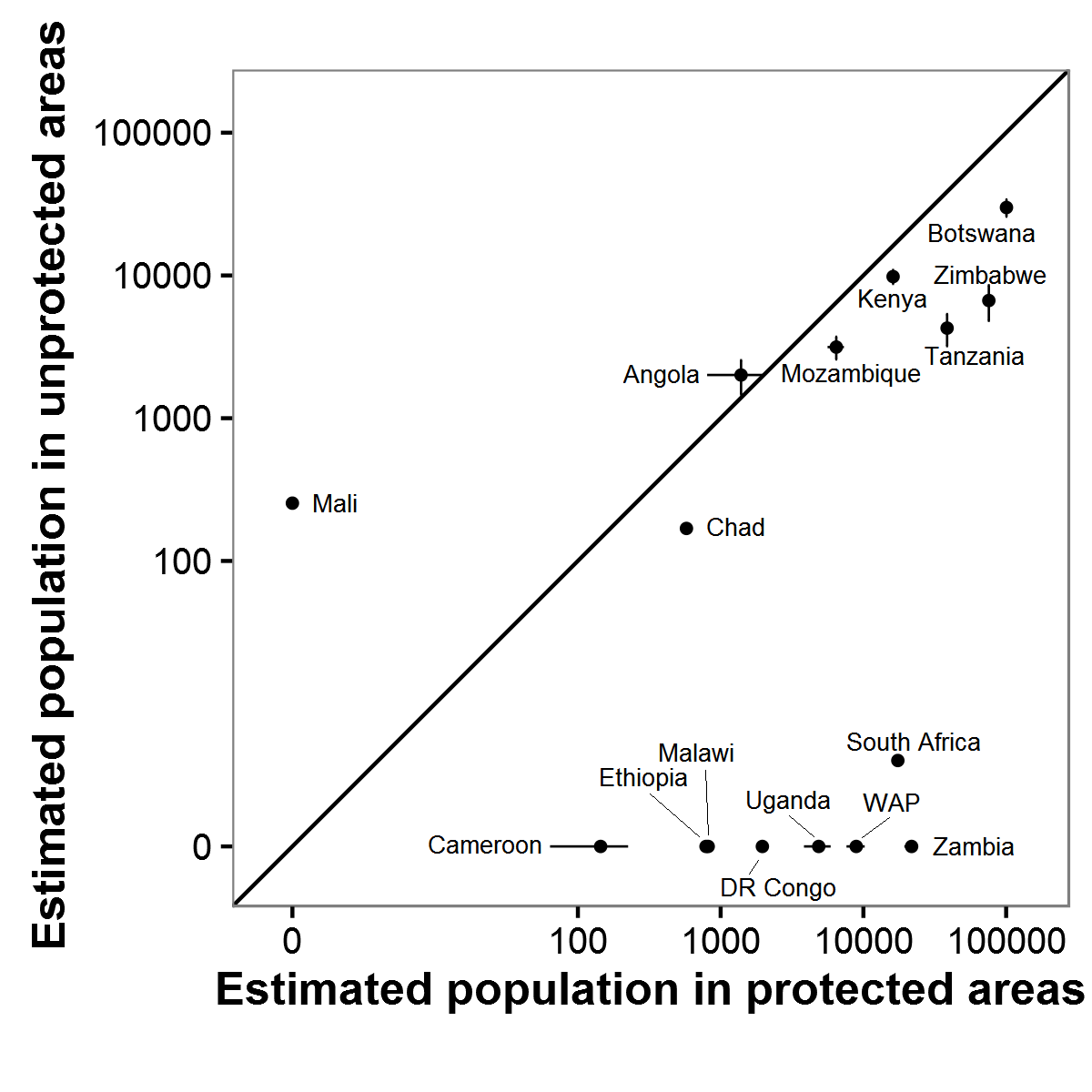

Supplement: Figure S5 — Error bars indicate ± 1 SE. [file peerj-04-2354-s005.png]

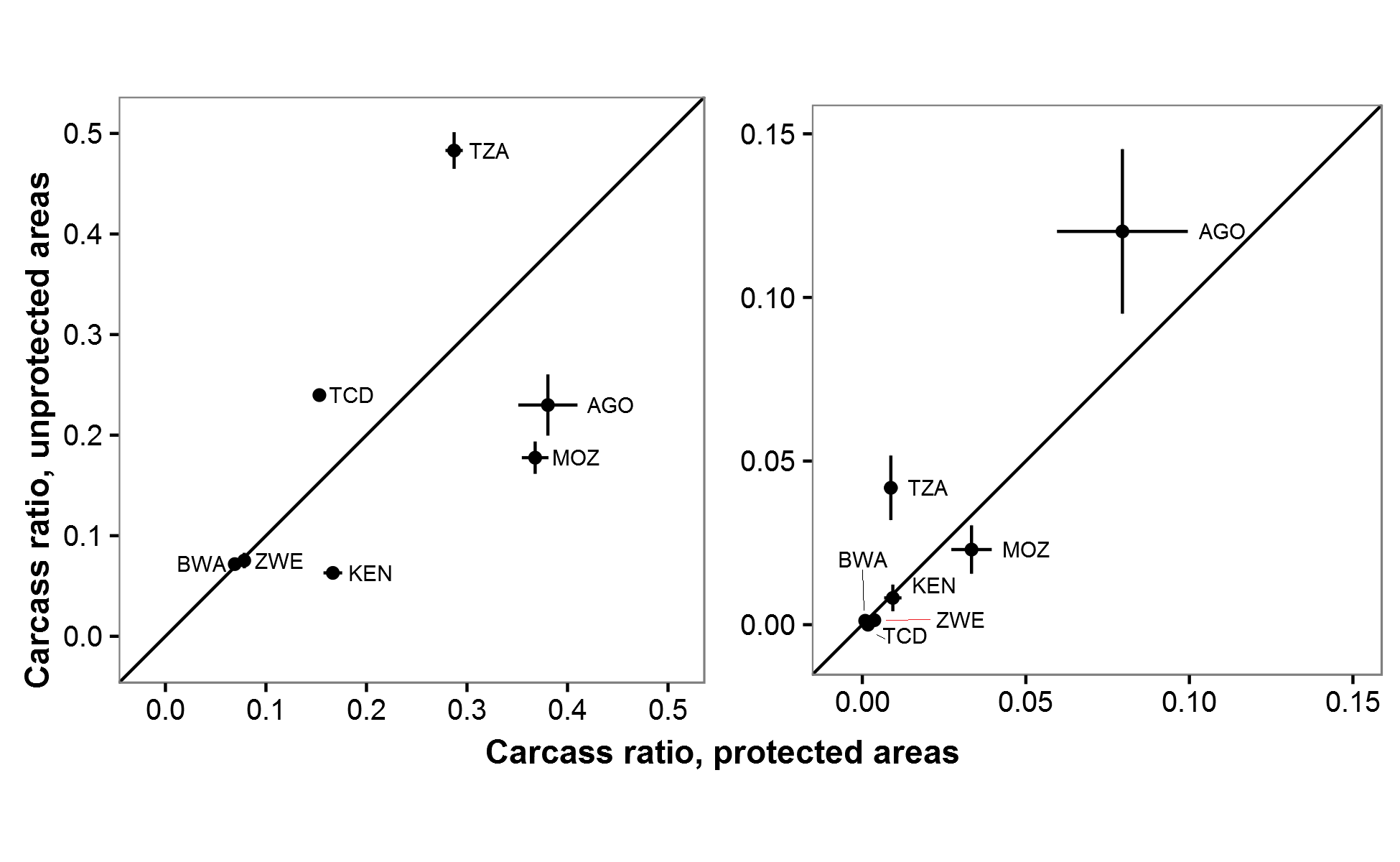

Supplement: Figure S6 — (A) ratios for all carcasses. (B) ratios for fresh carcasses. Only countries with elephant populations in both protected and unprotected areas are shown. Error bars indicate ±1 SE. Country codes: AGO–Angola, BWA–Botswana, TCD–Chad, KEN–Kenya, MOZ–Mozambique, TZA–Tanzania, ZWE–Zimbabwe. [file peerj-04-2354-s006.png]

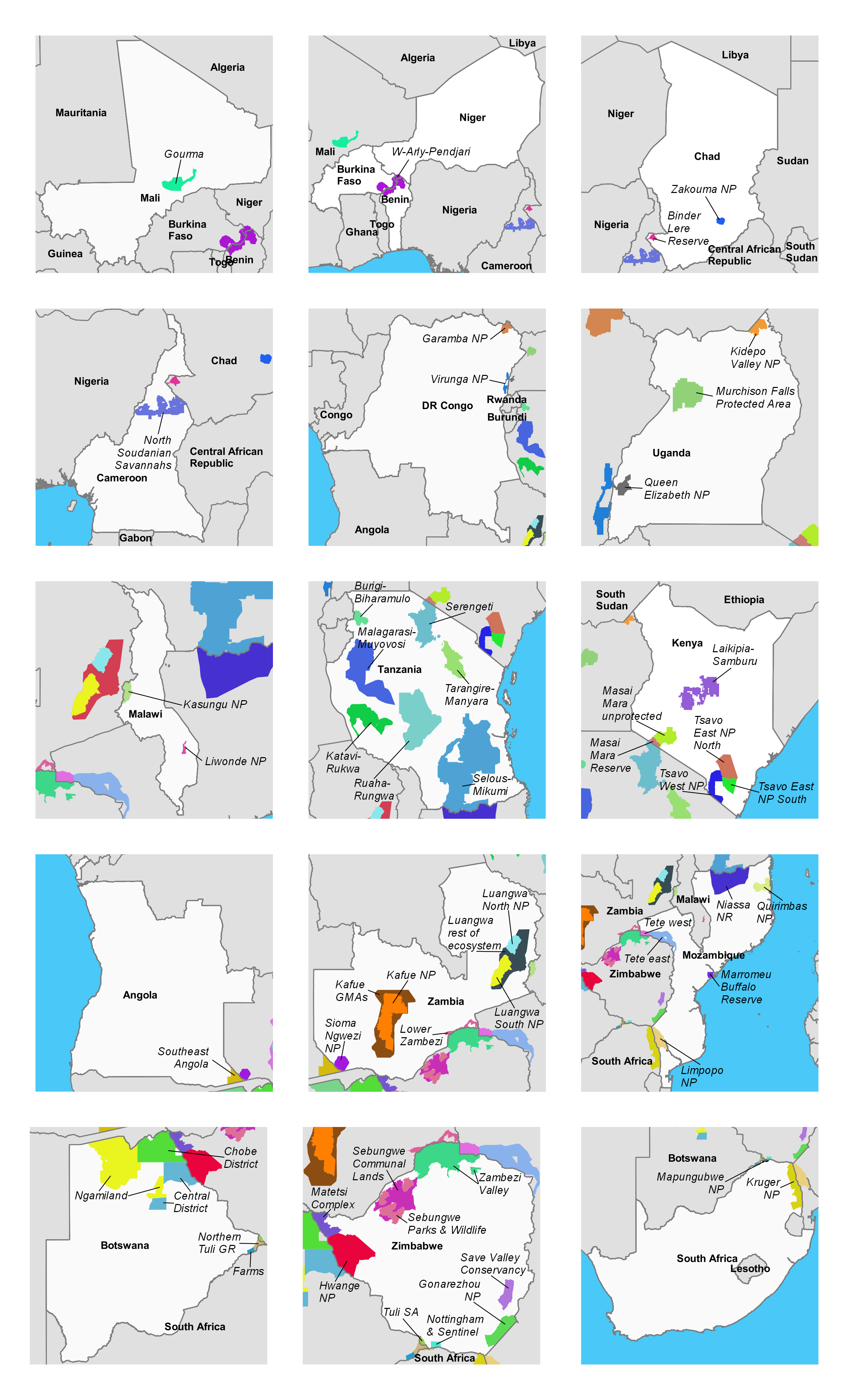

Supplement: Figure S7 — Unique colors signify different strata. Strata names are in italics. [file peerj-04-2354-s007.png]

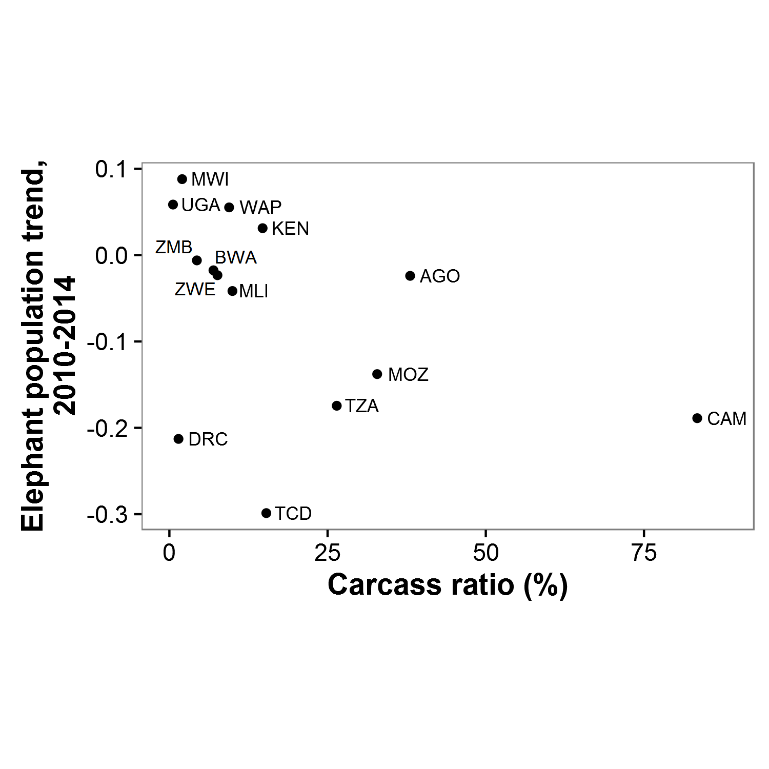

Supplement: Figure S9 — Country codes: AGO–Angola, BEN–Benin, BWA–Botswana, BFA–Burkina Faso, CAM–Cameroon, TCD–Chad, DRC–DR Congo, KEN–Kenya, MWI–Malawi, MLI–Mali, MOZ–Mozambique, NER–Niger, TZA–Tanzania, UGA–Uganda, ZMB–Zambia, ZWE–Zimbabwe. [file peerj-04-2354-s009.png]
