# Supplementary material for: Continent-wide survey reveals massive decline in African savannah elephants
Source: PeerJ. 2016 Aug 31;4:e2354. doi: 10.7717/peerj.2354 (PMC5012305; doi:10.7717/peerj.2354)

Historical estimates for a single country

Stratum	Year	Estimate	SE
A	1995	1,000	250
A	2002	1,300	300
**A**	2014	1,600	350
**B**	2001	500	0
B	2014	300	0

Generate 1,000 Monte Carlo samples based on each population estimate and SE.

Stratum	Year	Monte Carlo Estimate
A	1995	1,039
A	2002	1,602
**A**	2014	1,738
**B**	2001	500
B	2014	300

Monte Carlo replicate 1

...

Stratum	Year	Monte Carlo Estimate
A	1995	838
A	2002	768
**A**	2014	1291
**B**	2001	500
B	2014	300

Monte Carlo replicate 500

...

Stratum	Year	Monte Carlo Estimate
A	1995	797
A	2002	1,532
**A**	2014	1605
**B**	2001	500
B	2014	300

Monte Carlo replicate 1,000

GAM or Poisson regression to predict population size by year for each replicate.

Year	Predicted Population	SE
1995	1,437	233
...	....	....
2005	2,041	290
....	....	....
2014	2,110	339

...

Year	Predicted Population	SE
1995	1,168	227
...	....	....
2005	1,365	197
....	....	....
2014	1,571	313

...

Year	Predicted Population	SE
1995	1,083	185
...	....	....
2005	2,133	342
....	....	....
2014	1,940	313

Use Monte Carlo sampling to sample from the distribution of each prediction.

Year	Predicted Population
1995	1,492
...	....
2005	2,055
....	....
2014	2,044

...

Year	Predicted Population
1995	1,080
...	....
2005	1,622
....	....
2014	1,600

...

Year	Predicted Population
1995	1,271
...	....
2005	2,135
....	....
2014	1,683

Combine estimates by year and make inferences based on distribution of values.

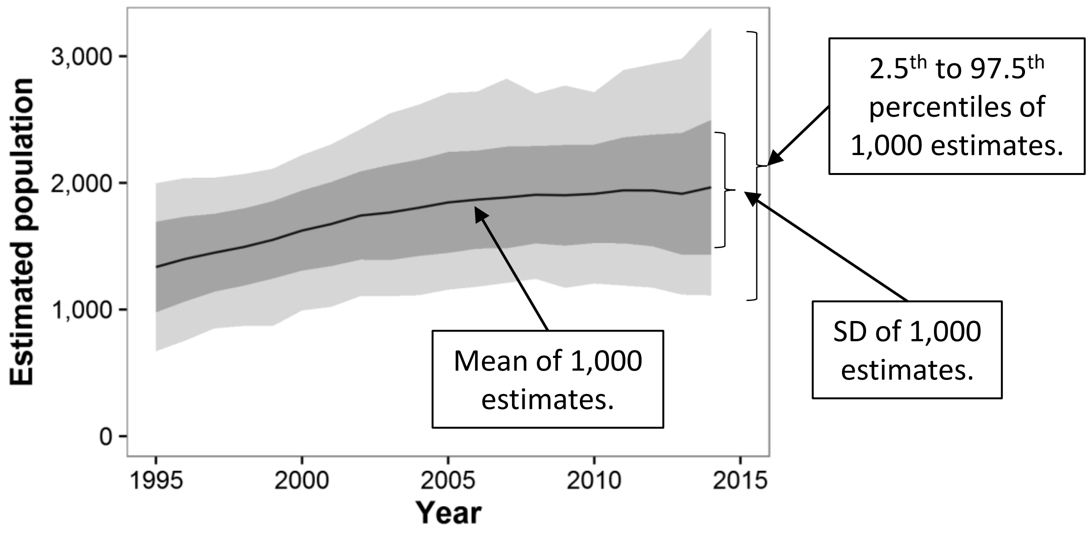

Supplement: Figure S3 — Hypothetical data (uppermost box) were created for this example, but numbers in boxes in subsequent steps are actual model output using the hypothetical data as input. [file peerj-04-2354-s003.pdf]
