# Supplementary material for: Continent-wide survey reveals massive decline in African savannah elephants
Source: PeerJ. 2016 Aug 31;4:e2354. doi: 10.7717/peerj.2354 (PMC5012305; doi:10.7717/peerj.2354)
Supplement: Figure S8 — Data points in 2014–2015 are from the GEC; earlier estimates come from published and unpublished reports and surveys. For Tsavo-Amboseli, the vertical line indicates an additional, non-GEC survey conducted in 2014. [file peerj-04-2354-s008.pdf]

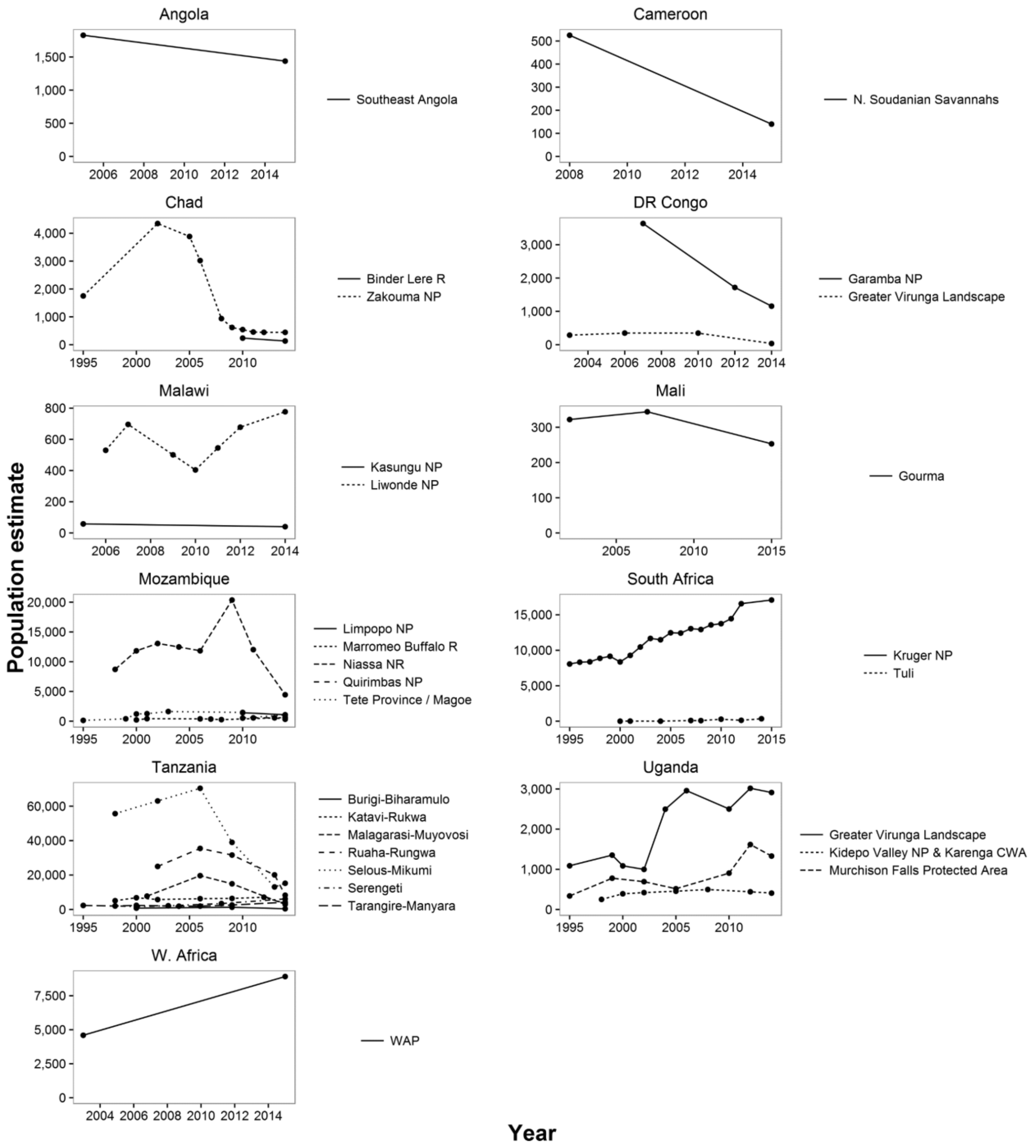
Angola
Southeast Angola
Cameroon
N. Soudanian Savannahs
Chad
Binder Lere R
Zakouma NP
DR Congo
Garamba NP
Greater Virunga Landscape
Malawi
Kasungu NP
Liwonde NP
Mali
Gourma
Mozambique
Limpopo NP
Marromeo Buffalo R
Niassa NR
Quirimbas NP
Tete Province / Magoe
South Africa
Kruger NP
Tuli
Tanzania
Burigi-Biharamulo
Katavi-Rukwa
Malagarasi-Muyovosi
Ruaha-Rungwa
Selous-Mikumi
Serengeti
Tarangire-Manyara
Uganda
Greater Virunga Landscape
Kidepo Valley NP & Karenga CWA
Murchison Falls Protected Area
W. Africa
WAP
Population estimate
Year

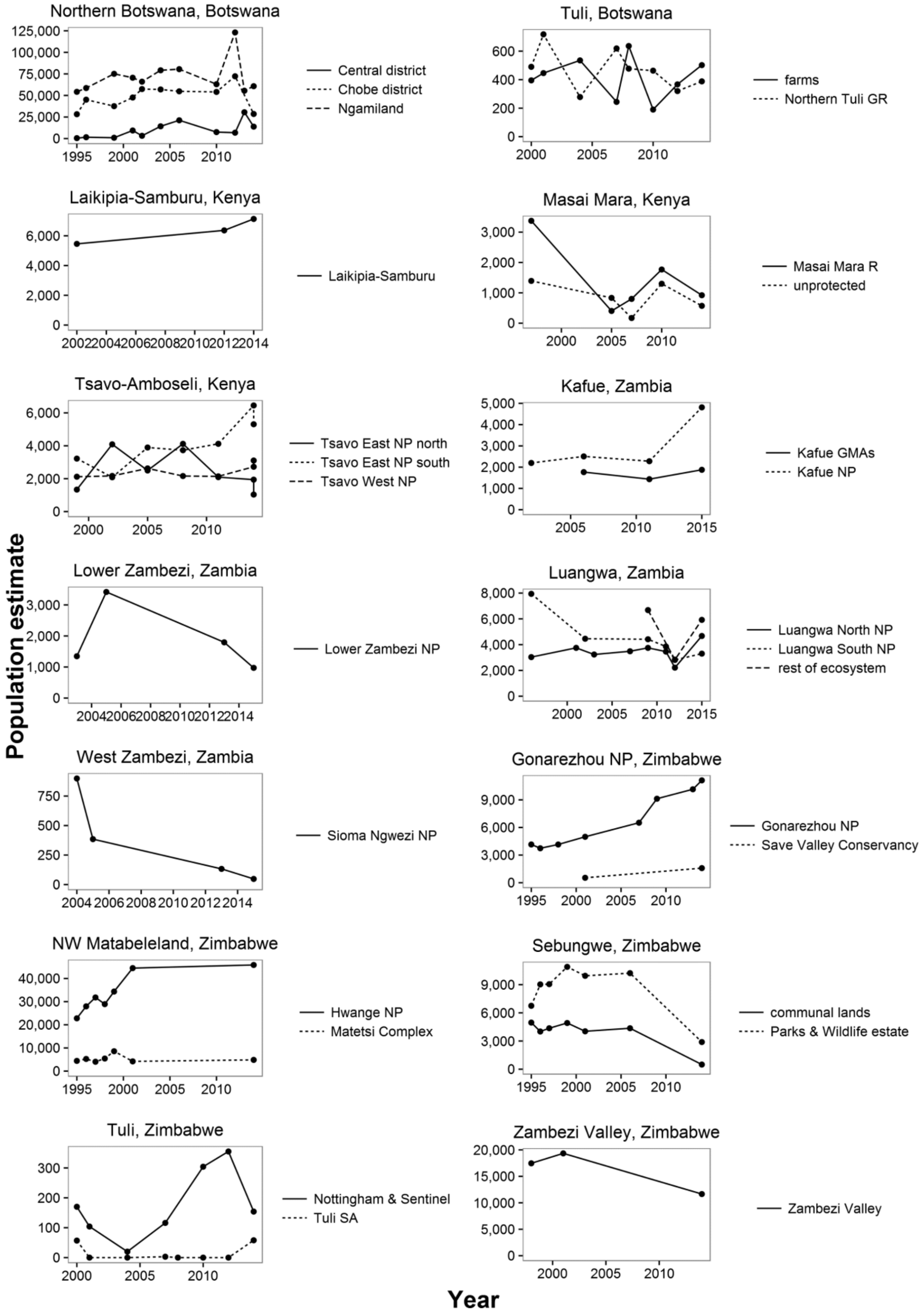

Northern Botswana, Botswana
Central district
Chobe district
Ngamiland
Tuli, Botswana
farms
Northern Tuli GR
Laikipia-Samburu, Kenya
Laikipia-Samburu
Masai Mara, Kenya
Masai Mara R
unprotected
Tsavo-Amboseli, Kenya
Tsavo East NP north
Tsavo East NP south
Tsavo West NP
Kafue, Zambia
Kafue GMAs
Kafue NP
Lower Zambezi, Zambia
Lower Zambezi NP
Luangwa, Zambia
Luangwa North NP
Luangwa South NP
rest of ecosystem
West Zambezi, Zambia
Sioma Ngwezi NP
Gonarezhou NP, Zimbabwe
Gonarezhou NP
Save Valley Conservancy
NW Matabeleland, Zimbabwe
Hwange NP
Matetsi Complex
Sebungwe, Zimbabwe
communal lands
Parks & Wildlife estate
Tuli, Zimbabwe
Nottingham & Sentinel
Tuli SA
Zambezi Valley, Zimbabwe
Zambezi Valley
Population estimate
Year
