# Supplementary material for: Continent-wide survey reveals massive decline in African savannah elephants
Source: PeerJ. 2016 Aug 31;4:e2354. doi: 10.7717/peerj.2354 (PMC5012305; doi:10.7717/peerj.2354)
Supplement: Table S1 — Abbreviations: R: reserve, NP national park, NR: national reserve, CWA: community wildlife area. [file peerj-04-2354-s010.docx]

| **Country** | **Ecosystem** | **Ecosystem area (km^2^)** | **Total transect length (km)** | **Area surveyed (km^2^)** | **Survey intensity (%)** | **Survey dates**  **(mo, yr)** | **Season (wet or dry)** |
| --- | --- | --- | --- | --- | --- | --- | --- |
| Angola | Southeast Angola | 43,459 | 9,443 | 3,386 | 8 | 11-12, 2015 | dry |
| Botswana | Northern Botswana | 98,307 | 29,900 | 12,166 | 12 | 7-10, 2014 | dry |
|  | Selebi-Phikwe | 1,974 | 1,586 | 1,974 | 100 | 2, 2015 | wet |
|  | Tuli | 1,318 | n/a | 1,318 | 100 | 8, 2014 | dry |
| Cameroon | N. Soudanian Savannahs | 20,598 | 4,955 | 1,509 | 7 | 4-5, 2015 | dry |
| Chad | Binder Lere R | 2,142 | 3,176 | 2,142 | 100 | 4, 2014 | dry |
|  | Mayo Kebbi Est & Chari Baguirimi Ele. Area | 3,312 | 5,261 | 3,312 | 100 | 4-5, 2014 | dry |
|  | N. Soudanian Savannahs | 1,144 | 457 | 134 | 12 | 4-5, 2015 | dry |
|  | Zakouma NP | 3,201 | 7,073 | 3,201 | 100 | 3-4, 2014 | dry |
| DR Congo | Garamba NP | 7,699 | 9,549 | 4,518 | 59 | 3-4, 2014 | early wet |
|  | Greater Virunga Landscape | 1,650 | 660 | 189 | 11 | 5-6, 2014 | dry |
| Ethiopia | Babile Ele. Sanctuary & SW Ethiopia | 14,691 | 3,553 | 14,691 | 100 | 4-5, 2014 | dry |
|  | NW Ethiopia | 18,100 | n/a | 18,100 | 100 | 4-5, 2014 | dry |
| Kenya | Laikipia-Samburu | 20,008 | 23,878 | 5,688 | 28 | 4-6, 2015 | wet |
|  | Lamu | 15,363 | 16,437 | 15,363 | 100 | 6, 2015 | wet |
|  | Masai Mara | 8,680 | 9,721 | 8,680 | 100 | 5-6, 2014 | wet |
|  | Tsavo-Amboseli | 41,660 | 8,347 | 2,670 | 6 | 3, 2014 | dry |
| Malawi | Kasungu NP | 2,316 | 291 | 2,316 | 100 | 10, 2014 | dry |
|  | Liwonde NP | 675 | 1,009 | 675 | 100 | 10, 2014 | dry |
| Mali | Gourma | 3,944 | 3,096 | 3,944 | 100 | 6, 2015 | wet |
| Mozambique | Limpopo NP | 15,211 | 4,201 | 1,799 | 12 | 9-10, 2014 | dry |
|  | Marromeo Buffalo R | 2,307 | 1,159 | 438 | 19 | 11, 2014 | dry |
|  | Niassa NR | 48,838 | 12,161 | 5,091 | 10 | 10-11, 2014 | dry |
|  | Quirimbas NP | 14,903 | 3,647 | 1,538 | 10 | 11, 2014 | dry |
|  | Tete Province / Magoe | 19,818 | 9,581 | 4,101 | 21 | 10-11, 2014 | dry |
| South Africa | Kruger NP | 19,485 | 18,139 | 19,485 | 100 | 8, 2015 | dry |
|  | Tuli | 324 | n/a | 324 | 100 | 8, 2014 | dry |
| Tanzania | Burigi-Biharamulo | 4,713 | 1,749 | 596 | 13 | 9, 2014 | dry |
|  | Katavi-Rukwa | 19,953 | 5,982 | 1,775 | 9 | 9, 2014 | dry |
|  | Malagarasi-Muyovosi | 44,809 | 8,956 | 2,509 | 6 | 9, 2014 | dry |
|  | Ruaha-Rungwa | 50,368 | 9,522 | 2,871 | 6 | 11, 2014 | dry |
|  | Selous-Mikumi | 105,730 | 21,365 | 6,474 | 6 | 10, 2014 | dry |
|  | Serengeti | 23,634 | 26,473 | 23634 | 100 | 5-6, 2014 | wet |
|  | Tarangire-Manyara | 18,725 | n/a | 18,725 | 100 | 8-9, 2014 | dry |
| Uganda | Greater Virunga Landscape | 2,269 | 830 | 243 | 11 | 5-6, 2014 | dry |
|  | Kidepo Valley NP & Karenga CWA | 2,398 | 5,491 | 2,398 | 100 | 6, 2014 | dry |
|  | Murchison Falls Prot. Area | 6,271 | 2,620 | 1,948 | 31 | 5, 2014 | dry |
| W. Africa | W-Arly-Pendjari | 29,981 | 9,113 | 2,914 | 10 | 6, 2015 | dry |
| Zambia | Kafue | 44,985 | 9,680 | 3,338 | 7 | 9, 2015 | dry |
|  | Lower Zambezi | 2,528 | 849 | 265 | 10 | 9, 2015 | dry |
|  | Luangwa | 32,819 | 7,031 | 2,494 | 8 | 9, 2015 | dry |
|  | West Zambezi | 4,482 | 1,504 | 545 | 12 | 9, 2015 | dry |
| Zimbabwe | Gonarezhou NP & Save Valley Conservancy | 10,409 | 3,714 | 1,115 | 11 | 10-11, 2014 | dry |
|  | NW Matabeleland | 24,959 | 6,181 | 1,877 | 8 | 10, 2014 | dry |
|  | Sebungwe | 15,227 | 5,821 | 2,053 | 13 | 7-9. 2014 | dry |
|  | Tuli | 1,253 | n/a | 1,253 | 100 | 8, 2014 | dry |
|  | Zambezi Valley | 17,003 | 4,233 | 2,142 | 13 | 6-8, 2014 | dry |
