# Supplementary material for: Continent-wide survey reveals massive decline in African savannah elephants
Source: PeerJ. 2016 Aug 31;4:e2354. doi: 10.7717/peerj.2354 (PMC5012305; doi:10.7717/peerj.2354)
Supplement: Table S2 [file peerj-04-2354-s011.docx]

| **Country** | **Ecosystem** | **Stratum** | **Count type** | **Estimate** | **SE** |
| --- | --- | --- | --- | --- | --- |
| Angola | Southeast Angola | Luiana NP | sample | 1,389 | 586 |
|  |  | other areas | sample | 2,006 | 540 |
|  |  | Ecosystem total | sample | 3,395 | 797 |
| Botswana | Northern Botswana | Chobe FR | sample | 3,777 | 747 |
|  |  | Chobe NP | sample | 17,212 | 1,663 |
|  |  | Kasane FR | sample | 283 | 135 |
|  |  | Kazuma FR | total | 130 | 0 |
|  |  | Mak. & Nxai Pan NPs | sample | 2,242 | 447 |
|  |  | Moremi GR | sample | 10,027 | 665 |
|  |  | Sibuyu FR | sample | 2,118 | 704 |
|  |  | unprotected areas | samp. + tot. | 93,711 | 6,017 |
|  |  | Ecosystem total | samp. + tot. | 129,500 | 6,378 |
|  | Selebi-Phikwe | Ecosystem total | total | 61 | 0 |
|  | Tuli | farms | total | 502 | 0 |
|  |  | Northern Tuli GR | total | 388 | 0 |
|  |  | Ecosystem total | total | 890 | 0 |
| Cameroon | N. Soudanian Savannahs | Bouba N'djida NP | sample | 3 | 0 |
|  |  | other areas | sample | 140 | 81 |
|  |  | Ecosystem total | sample | 143 | 81 |
| Chad | Binder Lere R | Ecosystem total | total | 132 | 0 |
|  | Mayo Kebbi Est & Chari Baguirimi Elephant Area | Ecosystem total | total | 168 | 0 |
|  | Zakouma NP | Ecosystem total | total | 443 | 0 |
| DR Congo | Garamba NP | Ecosystem total | samp. + tot. | 1,924 | 148 |
|  | Greater Virunga Landscape | Virunga NP / Ecosystem tot. | sample | 35 | 27 |
| Ethiopia | Babile Elephant Sanctuary & SW Ethiopia | Babile Elephant Sanctuary | total | 36 | 0 |
|  |  | Mago NP | total | 32 | 0 |
|  |  | Omo NP | total | 411 | 0 |
|  |  | Ecosystem total | total | 479 | 0 |
|  | NW Ethiopia | Alatash NP | total | 0 | 0 |
|  |  | Dabus Valley | total | 0 | 0 |
|  |  | Chebera Churchura NP | total | 0 | 0 |
|  |  | Gambela NP | total | 302 | 0 |
|  |  | Kafta Sheraro NP | total | 8 | 0 |
|  |  | Ecosystem total | total | 310 | 0 |
| Kenya | Laikipia-Samburu | Buffalo Springs NR & Samburu NR | total | 507 | 0 |
|  |  | Kerio Valley NR | total | 309 | 0 |
|  |  | Marsabit NR / Park | total | 55 | 0 |
|  |  | Meru & Bisnandi NP | total | 659 | 0 |
|  |  | Mwea NR | total | 58 | 0 |
|  |  | South Turkana NR | total | 2 | 0 |
|  |  | unprotected/community | samp. + tot. | 7,058 | 786 |
|  |  | Ecosystem total | samp. + tot. | 8,648 | 786 |
|  | Lamu | Ecosystem total | total | 60 | 0 |
|  | Masai Mara | Masai Mara R | total | 876 | 0 |
|  |  | unprotected areas | total | 552 | 0 |
|  |  | Ecosystem total | total | 1,428 | 0 |
|  | Tsavo-Amboseli | Amboseli NP | sample | 1,146 | 588 |
|  |  | Tsavo East NP North | sample | 1,033 | 395 |
|  |  | Tsavo East NP South | sample | 7,970 | 1,135 |
|  |  | Tsavo West NP | sample | 3,527 | 529 |
|  |  | unprotected areas | sample | 2,147 | 756 |
|  |  | Ecosystem total | sample | 15,823 | 1,625 |
| Malawi | Kasungu NP | Ecosystem total | total | 40 | 0 |
|  | Liwonde NP | Ecosystem total | total | 777 | 0 |
| Mali | Gourma | Ecosystem total | total | 253 | 0 |
| Mozambique | Limpopo NP | Limpopo NP | sample | 1,081 | 327 |
|  |  | southern reach | sample | 173 | 132 |
|  |  | Ecosystem total | sample | 1,254 | 353 |
|  | Marromeo Buffalo R | Ecosystem total | sample | 606 | 307 |
|  | Niassa NR | Niassa NR | sample | 4,441 | 694 |
|  |  | southern extension | sample | 1,016 | 301 |
|  |  | Ecosystem total | sample | 5,457 | 756 |
|  | Quirimbas NP | corridor to Niassa NP | sample | 306 | 167 |
|  |  | Quirimbas NP | sample | 328 | 184 |
|  |  | Ecosystem total | sample | 634 | 249 |
|  | Tete Province / Magoe | Magoe NP | sample | 0 | 0 |
|  |  | other areas | sample | 1,654 | 428 |
|  |  | Ecosystem total | sample | 1,654 | 428 |
| South Africa | Kruger NP | Ecosystem total | total | 17,086 | 0 |
|  | Tuli | Mapungubwe NP | total | 344 | 0 |
|  |  | private reserves/farms | total | 3 | 0 |
|  |  | Ecosystem total | total | 347 | 0 |
| Tanzania | Burigi-Biharamulo | Ecosystem total | sample | 402 | 381 |
|  | Katavi-Rukwa | Katavi NP | sample | 3,128 | 992 |
|  |  | Lukwati GR | sample | 1,090 | 281 |
|  |  | Rukwa GR | sample | 122 | 67 |
|  |  | Nkamba-Lwafi | sample | 209 | 111 |
|  |  | other areas | sample | 1,189 | 899 |
|  |  | Ecosystem total | sample | 5,738 | 1,375 |
|  | Malagarasi-Muyovosi | Moyowosi GR | sample | 1,645 | 1,219 |
|  |  | Ramsar site | sample | 503 | 302 |
|  |  | Ugalla GR | sample | 660 | 281 |
|  |  | other areas | sample | 146 | 142 |
|  |  | Ecosystem total | sample | 2,953 | 1,308 |
|  | Ruaha-Rungwa | Itigi Thickets | sample | 228 | 226 |
|  |  | Kizigo GR | sample | 758 | 506 |
|  |  | Ruaha NP | sample | 4,210 | 1,164 |
|  |  | Rungwa GR | sample | 2,801 | 971 |
|  |  | unprotected areas | sample | 275 | 170 |
|  |  | Ecosystem total | sample | 8,272 | 1,623 |
|  | Selous-Mikumi | Mikumi NP | sample | 885 | 300 |
|  |  | Selous-Niassa Corridor | sample | 1,161 | 690 |
|  |  | Selous GR | sample | 11,419 | 1,566 |
|  |  | other areas | sample | 1,736 | 463 |
|  |  | Ecosystem total | sample | 15,217 | 1,800 |
|  | Serengeti | Serengeti NP | total | 5,160 | 0 |
|  |  | other areas | total | 927 | 0 |
|  |  | Ecosystem total | total | 6,087 | 0 |
|  | Tarangire-Manyara | Tarangire NP | total | 3,282 | 0 |
|  |  | other areas | total | 920 | 0 |
|  |  | Ecosystem total | total | 4,202 | 0 |
| Uganda | Greater Virunga Landscape | Queen Elizabeth NP / Ecosystem total | sample | 2,913 | 932 |
|  | Kidepo Valley NP & Karenga CWA | Karenga CWA | total | 214 | 0 |
|  |  | Kidepo Valley NP | total | 407 | 0 |
|  |  | Ecosystem total | total | 621 | 0 |
|  | Murchison Falls Prot. Area | Ecosystem total | sample | 1,330 | 441 |
| W. Africa | W-Arly-Pendjari | Ecosystem total | sample | 8,911 | 1,299 |
| Zambia | Kafue | Kafue GMAs | sample | 1,876 | 819 |
|  |  | Kafue NP | sample | 4,813 | 1,149 |
|  |  | Ecosystem total | sample | 6,688 | 1,411 |
|  | Lower Zambezi | Lower Zambezi GMAs | sample | 153 | 86 |
|  |  | Lower Zambezi NP | sample | 973 | 295 |
|  |  | Ecosystem total | sample | 1,125 | 308 |
|  | Luangwa | Luambe NP | sample | 54 | 39 |
|  |  | Luangwa GMAs | sample | 5,869 | 1,574 |
|  |  | Luangwa North NP | sample | 4,673 | 903 |
|  |  | Luangwa South NP | sample | 3,302 | 605 |
|  |  | Lukusuzi NP | sample | 0 | 0 |
|  |  | Ecosystem total | sample | 13,898 | 1,802 |
|  | West Zambezi | Sioma Ngwezi NP / Ecosystem Total | sample | 48 | 38 |
| Zimbabwe | Gonarezhou NP & Save Valley Conservancy | Communal Land | sample | 332 | 225 |
|  |  | Gonarezhou NP | sample | 11,120 | 1,382 |
|  |  | Malapati Safari Area | sample | 0 | 0 |
|  |  | Save Valley Conservancy | sample | 1,585 | 661 |
|  |  | Ecosystem total | sample | 13,037 | 1,548 |
|  | NW Matabeleland | Communal Areas | sample | 2,201 | 1,562 |
|  |  | Forest Areas | sample | 1,101 | 507 |
|  |  | Hwange NP | sample | 45,846 | 3,186 |
|  |  | Matetsi Complex | sample | 4,843 | 1,514 |
|  |  | Ecosystem total | sample | 53,991 | 3,891 |
|  | Sebungwe | All communal lands | sample | 497 | 204 |
|  |  | Chete SA | sample | 278 | 114 |
|  |  | Chirisa SA | sample | 1,200 | 385 |
|  |  | Chizarira NP | sample | 747 | 391 |
|  |  | Matusadona NP | sample | 669 | 128 |
|  |  | Sijarira FA | sample | 16 | 9 |
|  |  | Ecosystem total | sample | 3,407 | 610 |
|  | Tuli | Nottingham & Sentinel | total | 154 | 0 |
|  |  | Tuli Safari Area | total | 58 | 0 |
|  |  | Ecosystem total | total | 212 | 0 |
|  | Zambezi Valley | Charara SA | sample | 36 | 24 |
|  |  | Chewore SA | sample | 3,303 | 462 |
|  |  | Communal Lands | sample | 1,904 | 706 |
|  |  | Doma SA | sample | 153 | 89 |
|  |  | Hurungwe SA | sample | 2,698 | 582 |
|  |  | Mana Pools NP | sample | 2,984 | 447 |
|  |  | Sapi SA | sample | 578 | 182 |
|  |  | Ecosystem total | sample | 11,657 | 1,137 |
