# Supplementary material for: Continent-wide survey reveals massive decline in African savannah elephants
Source: PeerJ. 2016 Aug 31;4:e2354. doi: 10.7717/peerj.2354 (PMC5012305; doi:10.7717/peerj.2354)
Supplement: Table S3 — Rows in bold indicate r significantly >0; rows in italics indicate r significantly <0. [file peerj-04-2354-s012.docx]

| **Country** | **Start year** | ***r*** | **Lower 95% CL** | **Upper 95% CL** |
| --- | --- | --- | --- | --- |
| *Angola* | *2005* | *-0.02* | *-0.03* | *-0.02* |
| *Angola* | *2010* | *-0.02* | *-0.03* | *-0.02* |
| **Botswana** | **1995** | **0.02** | **0.01** | **0.03** |
| Botswana | 2005 | -0.002 | -0.02 | 0.02 |
| Botswana | 2010 | -0.01 | -0.05 | 0.02 |
| *Cameroon* | *2008* | *-0.19* | *-0.20* | *-0.18* |
| *Cameroon* | *2010* | *-0.19* | *-0.20* | *-0.18* |
| *Chad* | *1995* | *-0.12* | *-0.15* | *-0.07* |
| *Chad* | *2005* | *-0.26* | *-0.3* | *-0.21* |
| *Chad* | *2010* | *-0.06* | *-0.22* | *-0.01* |
| *DR Congo* | *2003* | *-0.13* | *-0.18* | *-0.03* |
| *DR Congo* | *2005* | *-0.15* | *-0.19* | *-0.10* |
| *DR Congo* | *2010* | *-0.20* | *-0.30* | *-0.13* |
| Kenya | 1997 | 0.01 | -0.01 | 0.02 |
| Kenya | 2005 | 0.02 | -0.002 | 0.04 |
| Kenya | 2010 | 0.02 | -0.01 | 0.05 |
| Malawi | 2005 | 0.02 | -0.01 | 0.05 |
| **Malawi** | **2010** | **0.11** | **0.02** | **0.18** |
| *Mali* | *2002* | *-0.02* | *-0.02* | *-0.02* |
| *Mali* | *2005* | *-0.03* | *-0.03* | *-0.03* |
| *Mali* | *2010* | *-0.04* | *-0.05* | *-0.03* |
| **Mozambique** | **1995** | **0.03** | **0.01** | **0.08** |
| *Mozambique* | *2005* | *-0.09* | *-0.12* | *-0.06* |
| *Mozambique* | *2010* | *-0.24* | *-0.33* | *-0.16* |
| **South Africa** | **1995** | **0.04** | **0.04** | **0.04** |
| **South Africa** | **2005** | **0.04** | **0.04** | **0.04** |
| **South Africa** | **2010** | **0.04** | **0.04** | **0.04** |
| *Tanzania* | *1995* | *-0.03* | *-0.05* | *-0.01* |
| *Tanzania* | *2005* | *-0.14* | *-0.18* | *-0.09* |
| *Tanzania* | *2010* | *-0.19* | *-0.25* | *-0.12* |
| **Uganda** | **1995** | **0.06** | **0.03** | **0.10** |
| Uganda | 2005 | 0.05 | -0.02 | 0.08 |
| Uganda | 2010 | 0.04 | -0.07 | 0.10 |
| **W. Africa** | **2003** | **0.06** | **0.05** | **0.06** |
| **W. Africa** | **2005** | **0.06** | **0.05** | **0.06** |
| **W. Africa** | **2010** | **0.06** | **0.05** | **0.06** |
| Zambia | 1996 | -0.02 | -0.04 | 0.001 |
| Zambia | 2005 | -0.01 | -0.05 | 0.01 |
| Zambia | 2010 | -0.01 | -0.04 | 0.05 |
| Zimbabwe | 1995 | 0.02 | 0.01 | 0.02 |
| Zimbabwe | 2005 | -0.01 | -0.03 | 0.002 |
| Zimbabwe | 2010 | -0.02 | -0.05 | 0.004 |
| **TOTAL** | **1995** | **0.01** | **0.01** | **0.02** |
| *TOTAL* | *2005* | *-0.04* | *-0.06* | *-0.03* |
| *TOTAL* | *2010* | *-0.08* | *-0.10* | *-0.06* |
