# Supplementary material for: Continent-wide survey reveals massive decline in African savannah elephants
Source: PeerJ. 2016 Aug 31;4:e2354. doi: 10.7717/peerj.2354 (PMC5012305; doi:10.7717/peerj.2354)
Supplement: Article S1 [file peerj-04-2354-s013.pdf]

Aerial Survey Standards and Guidelines
for the
Pan-African Elephant Aerial Survey
2014

Principal Investigator
March 2014

Table of Contents

Introduction 3

1 Guidelines 4

1.1 Comparability Between Surveys & choice of method 4

1.2 Orientation of flight lines 4

1.3 Use of technology to support data collection and validation 4

2 Standards 6

2.1 Overview 6

2.1.1 Design and setup 7

2.1.2 Daily evaluations 8

2.1.3 Post-census validation 9

2.2 Survey Design 11

2.2.1 Target Census Parameters – Sample Count 11

2.2.2 Consistency between Aircraft 11

2.2.3 Duration of the Census 12

2.3 Strip Width Setup and Radar/Laser Altimeter Calibration 12

2.3.1 Strip width setup 12

2.3.2 Radar altimeter function check 12

2.4 Research Team Selection and Training 13

2.4.1 Pilot Selection 13

2.4.2 Rear Seat Observers Training and Calibrating 13

2.5 In Flight Data Capture 14

2.5.1 General 14

2.5.2 Pre-Flight 14

2.5.3 Navigation (Pilot) 14

2.5.4 Front Seat Observer (FSO) 14

2.5.5 Rear Seat Observers (RSO) 15

2.6 Post-Flight Data Processing and Validation 16

2.7 Post-Census Data Validation 16

2.7.1 Flight Parameters 17

2.7.2 Observer Consistency 17

2.7.3 Census Parameters 17

2.8 Data Analysis 18

2.9 Reporting 19

2.9.1 Narrative Report 19

2.9.2 Datasets 20

3 References 21

Introduction

The following guidelines and standards, which are largely based on the 2012 MIKE 2.0 aerial survey standards, were discussed with aerial survey researchers during the January 27th – 29th 2014 Pan-African Elephant Aerial Survey (PAEAS) meeting sponsored by Elephants Without Borders and Paul G Allen and held in Kasane, Botswana. The intent of this document is to provide the PAEAS Technical Advisory Team (TAT) with a reference to review survey plans and technical reports. It also serves to guide country survey teams to finalize their survey plans and ensure consistent protocols relating to:

1. Field methods;
2. Planning and reporting obligations; and
3. Standards of review for the PAEAS Technical Advisory Team (TAT) please refer to the synopsis document.

This document is divided into two sections:

Guidelines: suggestions for choice of method and survey design.

Standards: minimum requirements that will be requirements for funding contracts.

It is understood that some country surveys being flown as part of the PAEAS cannot be rigidly subjected to "one-size-fits-all" requirements. Where the Standards cannot be adhered to due to changing field conditions, we encourage country survey teams to communicate with the TAT to seek approval for significant deviations from the Standards.

1 Guidelines

1.1 Comparability between surveys and choice of method

The PAEAS strongly supports transect sample counts as a standardised method. As per the MIKE standards, total counts may be chosen where needs dictate (small areas and populations, highly clumped populations where large groups represent a large proportion of the population), and block counts where transect flying is unfeasible.

Where surveys have been regularly flown, sampling coverage (survey boundary) should follow that of previous surveys, adhering to the standards presented herein.

In comparison to previous surveys conducted if it deemed that conducting a total count would be more beneficial than a prior sample count, the country survey team is to submit justification for the proposed change in survey design and the implications of those changes.

1.2 Orientation of flight lines

The three factors to consider when selecting the orientation of flight lines are (a) to reduce census variance (and thus increase census precision) by maximising the number of flight lines (sample units) flown at any given sampling intensity; (b) to avoid up-sun / down-sun observer bias (thus improving census accuracy); and (c) to cut across major ecological gradients (e.g. major river lines) which in turn reduces census variance.

There are no absolute rules to determine which of these factors is the most important, each census will be unique and each decision will be a compromise. However, all things being equal East-West is preferred.

1.3 Use of technology to support data collection and validation

It is desired that each research team use interior mounted cameras and voice recorders to support collection and validation of data. The following equipment specifications are provided as guidelines to the country survey teams to consider:

- Cameras: Aerial photography is one tool frequently used to improve the precision and accuracy of wildlife surveys. Two digital cameras (EOS 7D Canon, 18 mega pixel or equivalent) should be fixed to specially adapted mounts on each window of the plane. The centre of the lenses must correspond to marks on the plane window used to help observers keep their eyes at a consistent height for each observation. The focal length of the lens must be adjusted to incorporate the full counting strip width, providing a consistent viewing angle. If any animal group is too large for all the individuals within it to be counted, a digital photo must be taken (typically any group higher than 5 animals). These photos are used to verify herd size and the sighting of herds within the interval defined by the wands. The components of the camera system should consist of two cameras with 20-mm wide-angle lenses, camera backs with time code generators, remote switches and two window camera mounts. A camera must be mounted on each side of the plane. The cameras provided high-resolution photos so that animals could be more accurately counted during subsequent analyses. A GPS time code and date must be recorded to the second for every frame exposed.
- Voice recorder: All four crew members should be able to communicate efficiently through a four-way intercom headset receiver box. Observers in the plane call out their

observations into a recorder, which should have a 4 GB memory card/storage. At the end of each day, recordings are played back on a laptop computer to verify hand-recorded data.

Further, the TAT acknowledges the advancements in precision that can be gained by using laser altimeters versus radar altimeters and global position systems. As such, please consider the following equipment specs:

- Laser altimeter: Country survey teams are encouraged to use the ILM laser module provided by Aglaser (www.aglaser.com), which comes with the navigation display (~$3000). It's offered standard with the 150 meter module, but would ask for them to supply it with a 500 meter module (http://mdl-laser.com/en/ilm-500--15114). This instrumentation should be complemented with the standard l Bendix King radar altimeter.
- GPS: A Garmin Cx60 hand held GPS is recommended to store GPS location of observations and tracklog of the plane's flight path. The GPS has a strong external antenna, and capable of storing 1000 location points.

The PAEAS team acknowledges that there are concerns about the adoption of cameras with respect to observer distraction and consequently detection of animals.

1. Training can be provided by PAEAS for teams unfamiliar with camera use, and extra flight hours (typically 2 x 2.5 hour sessions) are encouraged as part of the budgeting for surveys.
2. Experimental protocols involving the alternating use / non-use of cameras for each observer would be supported for initial adoption, though a good experimental design would need to be provided.

2 Standards

2.1 Overview

The following tables summarise the three stages of required setup, validation and reporting protocols for PAEAS surveys. The implementation of all of these components are required to be reported in the proposal, in the monthly reports and in the final report.

I. Pre-survey design and setup: the design of the survey (block and transect layouts, equipment and training).
II. Post-flight evaluation: primary validation done on a daily basis, designed to catch gross errors in performance by crews, and allowing the team to drop transects (or single strips) and re-fly sections where necessary.
III. Post-census (secondary) validation: while this is a requirement, the TAT recognises that the more complex of the validation protocols may be difficult to complete if country survey teams are unfamiliar with the protocols (or busy with finishing other surveys); at a minimum the data provided together with the report will allow the TAT to do secondary validation afterwards.

2.1.1 Design and setup

Section	Item	Target
Operational Targets	• Height AGL sample counts	300 feet
	• Height AGL total counts	300 feet
	• Strip Width	150m preferred, 200m (per side) if flown by previous surveys
	• Ground Speed	160 kph - 180 kph
	• Time of Day	Morning: 45 minutes after sunrise, no later than ~1100<br>Afternoon: Up to 45 minutes before sunset, no earlier than 1500.
	• Session Length	3.0 hours on-the-line (3.5 maximum)
	• Uninterrupted transect length (before a break)	25 minutes (35 minutes as absolute maximum)
	• Rest days	Every fifth day
	• Delay between censusing neighbouring blocks	Maximum 2 days
Equipment	• Photography	Window mounted, digital 35mm SLR camera with minimum 14 megapixels, and cable shutter release.
	• Demarcating strip width (rods / wands)	Adjustable, rigid (fibreglass) rods attached to wing struts
	• Aircraft	High wing a/c with wing struts.
Data Capture Protocols	• Calibration	Full strip width / radar (or laser) altimeter / pressure altimeter calibrations.<br>Ideally use large numbered ground markers.
	• Subunit method	2.5 km subunits, FSO calls out to RSO, each RSO has individual tape recorder<br>IF RSOs call out to FSO, then full cockpit tape recorder required as well<br>FSO records height AGL and GS once per subunit
	• Call-out method	RSOs call out to FSO, FSO creates GPS waypoint for each observation, full cockpit tape recorder<br>FAO records height AGL and GS for each observation, AND every 30 seconds.
Crew Preparation	• All observers	Test of visual acuity and colour blindness<br>Training photos for identification and counting in census habitat
	• Naive observers (no previous experience, or no flights within previous 12-18 months)	Training photos for identification and counting in census habitat<br>3 * 2.5 hour training flights (as per TAWIRI protocols (Norton-Griffiths-Frederick 2013)
	• Pre census familiarisation flight	2.5 hour familiarisation flight<br>Full census protocols<br>Full post- flight performance evaluation

2.1.2 Daily evaluations

Item	Statistics	Action
Data Quality	• Trap coding errors and transcription errors;<br>• Validate all photo counts;<br>• Trap outliers	
Height AGL	• Frequency histogram (10 feet intervals);<br>• Calculate mean, standard deviation and total observations<br>• Compare mean Height AGL against target	Target height +/- 10% SE allowable; check with pilot when outside bounds, change pilot if consistently >10% variation.<br>Re-fly transects where necessary.
Ground Speed	• Frequency histogram (10 kts intervals);<br>• Calculate mean, standard deviation and total observations<br>• Compare mean GS against target	Ground speed should be no less than 160 km h^{-1} and NOT exceeding 190 km h^{-1} for entire transects or for more than 30 seconds in a given transect.<br>Re-fly transects where necessary.
Ground Speed On Reciprocal Headings	• ANOVAR of ground speed (continuous variable) against reciprocal headings (e.g. E - W as categorical variable)	Discuss with pilot if ground speed significantly different on reciprocal headings; re-fly transects if necessary
Observer Consistency	• OBSERVED number (and %) of groups and total individuals (for important species) seen by Left and Right observer;<br>• Correct for strip width to give EXPECTED values;<br>• χ^2 = (OBS-EXP)^2/EXP for each observer;<br>• $\Sigma\chi^2$ for difference between pairs	45% - 55% OK; 40% - 60% worth looking into; <40% and >60% indicates a significant problem.

2.1.3 Post-census validation

Each aircraft

Item	Statistics	Action
Height AGL (HAGL)	• Frequency histogram across all flights; mean and standard deviations;<br>• ANOVA of HAGL (continuous variable) against flight number and transect number (categorical variables);<br>• Test individual mean HAGL for census, flights and transects against target HAGL	Determine if there is a large problem, namely if any flights / transects are so clearly out of bounds that action must be taken.<br>1. Remove those flights from the analysis (adjusting area/strata where necessary), or<br>2. Report likely nature and direction of resulting bias if transects are retained.
Ground Speed (GS)	• Frequency histogram across all flights; mean and standard deviations;<br>• ANOVA of GS (continuous variable) against flight number and transect number (categorical variables);<br>• Test individual mean GS for census, flights and transects against target GS;<br>• ANOVA of GS against reciprocal flight direction	

Observer pairs

Left / Right consistency	• For each observer, total the number of groups recorded and the total animals counted (corrected by photo-counts);<br>• Correct for strip widths to give expected values;<br>• χ^2 to test for statistical difference	45% - 55% OK;<br>40% - 60% worth looking into;<br><40% and >60% H-WHAP[1]<br><br>If # groups different then probable observer problem<br>If # groups not different but # counted different, then test photo counts and camera operation, also camera resolution and clarity of window Perspex

[1] "Houston – we have a problem."

Observer / flight interactions

Height AGL	• Divide height flown into quartiles, test % groups and % counted in each quartile	We hope to demonstrate excellent census design by failing to find any significant gross errors or discrepancies.<br><br>Minor differences or discrepancies which will have a small effect relative to the sampling error will feed back into the census design.<br><br>Major differences and discrepancies of a magnitude that remedial action is necessary, such as excluding part of the census data.
Reciprocal Headings	• Test # groups and # counted on reciprocal headings	
Ground Speed (GS)	• Divide GS into quartiles, test % groups and % counted in each quartile	
Time of Day	• Compare % groups and % numbers against % sampling effort in each hour of the day	
Fatigue	• Type 1: Divide transects into successive 15 minute intervals; test # groups, # counted and % left/right against each successive interval<br>• Type 2: Test # groups, # counted and % left/right, against successive counting hours since take off<br>• Type 3: Test # groups, # counted and % left/right against successive census days.	

Aircraft comparisons

Height AGL	• ANOVAR of height AGL against each aircraft (categorical variable)	As per table above, check and report major inconsistencies and likely scale of bias.
Ground Speed (GS)	• ANOVAR of GS against each aircraft (categorical variable)	
Interleaved Flight Lines	• Compare % groups and % numbers against % sampling effort by each aircraft in overlap area;<br>• Compare population estimate and variances	
Double count of sample blocks or strata	• Compare % groups and % numbers against % sampling effort by each aircraft in overlap area;<br>• Compare population estimate and variances	

2.2 Survey Design

2.2.1 Target Census Parameters – Sample Count

Based on previous experience, the following target census parameters are recommended for all sample counts:

- Height Above Ground: 300 feet +/- 30' SE.
- Ground Speed: 160 - 180 kph (86 - 97 knots), not to exceed 190 kph.
- Observer Strip Width: min. 150, max. 200 meters (either side of the aircraft)
- Search rate: 1-1.5 km^2h^{-1}.

These parameters must be monitored continuously within each flight, and analysed at the end of each flight. This allows pilot performance to be continuously assessed, both within and between flight crews. Pilots who are unable to perform consistently (height AGL > +/- 30' SE on average, ground speed regularly more than 190 km/h) will be dropped from the survey.

2.2.2 Consistency between Aircraft

While it is often acknowledged that pilots do vary in their proficiency and concentration, it is not often realised that different aircraft provide quite different observation platforms and therefore create quite different observer bias. When more than one aircraft is used for a survey it is important to ensure that each is equally efficient, and consistent, in terms of meeting the target census parameters and in observing and counting animals. The following are factors that can influence aircraft consistency:

- Type of aircraft;
- Survey speed and altitude control;
- Communications between crew members; and
- Cockpit noise, comfort and visibility

Each of the above need to be noted in the survey plan and technical reports and any likely impact to the consistency between aircraft should be noted and figured into the data analysis plan.

It is required that:

- Interleaved Flight Lines
  - Where two or more aircraft are used, fly interleaved flight lines so that each is in principal surveying "the same" area as the others.
  - On the initial coverage at 10km intervals, and for the 5km and 2.5km infilling, each aircraft would fly every other flight line (two aircraft) or every third flight line (3 aircraft).
- Census Parameters
  - Tracklogs and height readings must be download, and analysed, daily after each flight. It is then straightforward to monitor pilot performance and ensure consistency both within and between pilots.
- Animal Counts
  - The number of observations and the number of animals counted by each observer in each aircraft must be analysed after each flight. It is then straightforward to monitor observer performance and ensure consistency between observers within an aircraft (are both capturing "the same" amount) and between aircraft (is each aircrew capturing "the same" amount).

2.2.3 Duration of the survey

While any census should be completed as "quickly as possible" to minimise problems from animal movements, we do know that fatigue radically effects the performance and consistency of census aircrew, both pilots and observers alike.

Three types of fatigue are recognised:

- Type 1: on very long flight lines, fewer animals are counted towards the end of the flight line than at the beginning;
- Type 2: as the day wears on, fewer animals are counted and pilot performance degrades; and
- Type 3: as the census wears on, fewer animals are counted and pilot performance and consistency is quite poor.

It is required that:

- No single flight line should be more than 30 minutes in length. If longer, then the aircraft must break off the census for five minutes before taking up the line again.
- Each day, no more than 4.5 hours should be spent "on-the-line" observing.
- If two flight sessions are planned during one day, no more than 3.5 hours maximum (no longer than 3 strongly advised) should be spent on the first flight, no more than 2.0 hours spent on the second flight with a substantial break in between flights.
- A complete rest day for each aircrew on every 5th day.

These standards are known to minimise fatigue and if followed no fatigue effect can be discerned in the data. If the country survey team desires a different census duration plan justification needs to be provided and will be reviewed by the TAT.

2.3 Strip width setup and radar/laser altimeter calibration

2.3.1 Strip width setup

The observer sample strip width calibration must be done before the census, and routinely during the census, using standard methods.

- Tape markers to be placed in windows of aircraft to allow consistency of observer head position.
- Calibrations of observer strip width against radar altimeter must be done according to Frederick, et al 2010 - R squared of 0.9, intercept to +/- 20 metres, nominal strip width (at target altitude) to within +/- 20m of 150m.
- One calibration pass to be made over the airstrip on each take-off and landing for each observational (mission) session, at random height between 150’ and 350' AGL (to be used in validation).

2.3.2 Radar altimeter function check

It is recommended that all research teams utilize a laser altimeter to overcome the challenges noted by the research teams with radar altimeters. Resources are available to help locate the appropriate laser altimeter.

The function of both laser and radar altimeters (‘AGL altimeters’) must be checked, following these procedures:

- On each pass over the airstrip during calibration, the pilot should stabilise the aircraft at a target barometric pressure height (field elevation + 300 feet, for example);
- As the aircraft passes over the runway and the observers count the markers, the FSO records the AGL height, and the pilot calls out the actual barometric altimeter height.
- During calibration calculations, the regression of AGL and barometric altimeters should have an intercept of the field elevation and a slope of 0.95-1.07. Outside of these ranges (i.e. 1.13) the radar altimeter is most likely faulty and needs to be calibrated by an avionics professional.
    - Where it is impossible to return the AGL altimeter for calibration, the slope of the AGL/ barometric altimeter relationship must be checked daily, and if a deviation of > 0.05 is seen the altimeter must not be used. (Note that repeat passes should be made to confirm this).

Initial tests must be done BEFORE the aircraft leaves its home base:

- At home base, the pilot takes off and sets the pressure altimeter to an even-thousands (i.e. 0 feet, 1,000 feet, 4,000 feet, etc) on rotation;
- The pilot should make a minimum of four passes, flying the runway heading, at target **pressure** heights of 200, 300, 400 and 500 feet, with the co-pilot writing down three radar alt readings along the runway heading while the pilot stabilises the pressure altimeter.
- The pressure height differences and radar height differences should match nearly exactly (i.e. going from 3,200' pressure to 3,500' pressure should show an increase in radar altimeter readings of 300' as well).
- If the graph does not show a straight relationship the pilot must report this to the client immediately, and the aircraft remain at base until checked by an avionics technician (but see note above).

2.4 Research Team Selection and Training

2.4.1 Pilot selection

While the MIKE 2.0 standards indicate that pilots must have 1000 hours of flight time before they can conduct an aerial survey, the PAEAS is requiring only that the pilot has experience with both bush flying and flying at low levels and the necessary license and certifications to fly the country survey team aircraft. The country survey team must feel comfortable that their pilot will be able to adhere to the target census parameters and be able to keep the research team out of harm's way.

2.4.2 Rear Seat Observers training and calibrating

- Rear seat observer performance must be checked and validated before, during and after surveys. Only observers with documented performance may fly: Each observer to pass certification test (species ID and spotting from aircraft) as per TAWIRI certification workshop in 2012 (Annex within Norton-Griffiths-Frederick 2013);
- Each observer to have minimum 3 sessions of training flights, confirming reliability with ID and multi-species ID, fatigue issues, and photos of herd sizes greater than 5. (Annex within Norton-Griffiths-Frederick 2013)

Training will be offered on request from PAEAS for all rear seat observers who need first time training and for those who need refresher training. If rear seat observers will not attend this offered training, the country survey team must provide documentation of the training observers have received and their ability to be compliant with the standards set forth in this document.

2.5 In Flight Data Capture

While the standard protocols (per MIKE 2.0) will be followed for capturing front seat observer (FSO) and rear seat observer data, the following specific procedures are recommended.

2.5.1 General

- All navigation data, especially the way points identifying the start and end points of each flight line, must be pre-programmed into the a/c GPS.
- The track log must be set to a minimum of one reading per second (or if using a Garmin to the “automatic, often” setting).
- Each flight (for each aircraft) must have a unique i/d.
- Each flight line must have a unique i/d.

2.5.2 Pre-Flight

- A pre-flight briefing for each flight crew is essential so that all crew members are aware of exactly what each flight will entail.
- Both RSO cameras, and that of the FSO if used, must be calibrated with the a/c GPS by photographing the a/c GPS time readout before take-off.

2.5.3 Navigation (Pilot)

- Flight line navigation will be primarily by reference to the a/c GPS.
- The pilot will continuously monitor ground speed and cross-track by reference to GPS readouts, and height above ground by reference to the radar altimeter.

2.5.4 Front Seat Observer (FSO)

- FSO data will be recorded, by hand, onto prepared data sheets.
- Flight Data: for each flight, the FSO will record:-
    - the date, aircraft registration and airstrip;
    - the i/d of the pilot and the FSO; the i/ds and seating positions of both RSOs; and the flight i/d; and
    - the take-off and landing times.
- For each flight line: the FSO will record:
    - the flight i/d;
    - the flight line i/d
    - the start and end times, and the direction of flight; and
    - will call out to the RSOs the start and finish of each flight line.
- For each subunit: the FSO will:-
    - identify the start of each successive subunit along each flight line by reference to the a/c GPS;
    - call out the start and subunit number to the RSOs;

- record once within each subunit, without the knowledge of the pilot, the height above ground level (from radar/laser altimeter) and the ground speed (from GPS readout); and
- record any other environmental / landuse or other data agreed to by the census organisers.

2.5.5 Rear Seat Observers (RSO)

- All RSO data will be recorded onto hand held tape recorders.
- For each flight, the RSOs will record the date, flight number and their position in the aircraft.
- For each flight line, the RSOs will record the flight line number.
- For each subunit, the RSOs will record the subunit number.
- Standard protocols will be used to record species identification, estimates of group size, and photos taken, and include but not restricted to the following:
  - Each flight - Aircraft registration and model, crew names, time of take-off and landing, survey name, date, tracklog (recorded as a line and point);
  - Each sampling unit - Survey name, stratum name, sampling unit name/number, time of start, time of finish, positions of start point and end points (decimal degrees), height in feet above ground every 30 seconds, waypoints of each observation, weather;
  - Each sighting - species/observation, number seen, left or right of aircraft, GPS location, stratum name, altimeter reading, notes;
  - Species/observations that must be recorded -
    - Carcasses
      - Carcass 1
      - Carcass 2
      - Carcass 3
      - Carcass 4
      - Remark whether tusks present in carcass and any unusual abnormalities, e.g., carcass covered in bushes, appearance that tusks might have been chopped out, carcasses in close proximity to each other, etc.
    - Elephants in family group
    - Elephants in bull group
    - Other species (wild and domesticated), larger than 15 kgs and any other species deemed necessary by wildlife authorities as well as all anthropogenic disturbances.
- If photography is used observations of animal groups should be taken with window mounted cameras. Where cameras have not integrated into prior surveys, the country survey team will note the transition plan they are deploying to ensure 1) consistency between surveys and 2) integration of this technology in future surveys.
- Anthropogenic features must be recorded spatially in a GPS during the survey as well as all domestic animals (e.g. cattle, sheep, goats and donkeys). Any human disturbance/activity (inside (RSO) and outside of the counting strip by the FSO) on habitats in the survey area must be noted and spatially referenced e.g. poaching camps, logging, mines and camps/lodges. Agricultural fields (subsistence and commercial) must also be recorded, and all human settlements should be categorised into huts (corrugated iron or grass thatched

roofing), village or town. Same methods should be used to estimate their abundance and illustrate their location/distribution.

2.6 Post-Flight Data Processing and Validation

After each flight, all data must be downloaded from the a/c GPS (track logs), transcribed (from the RSO tape recorders) and catalogued. These raw data must then be analysed to identify and rectify any problems with consistency or errors in the way the census is being carried out.

Data downloading, processing and validation must be completed daily for each flight.

- Navigation:
  - For each individual flight, the track logs must be analysed for ground speed and cross-track error, and the FSO records for heights above ground level every 30 sec.
  - Pilot performance must be compared against census targets and discussed with the pilot.
  - Pilot performance should be analysed on both a flight-by-flight and a cumulated basis.
- FSO and RSO Data:
  - The consistency of the FSO and RSO data must be thoroughly checked, e.g. dates, a/c, crew, flight number, flightline number, flight direction, number of subunits etc.
- RSO Data:
  - The key parameters here are the numbers of observations and the (rough) numbers counted by each observer for each species.
  - Ideally, each observer should capture "the same" amount of data, but this will of course vary from flight line to flight line.
  - What is being looked for here are gross deviations from expected values which will indicate that the observers are not performing in a consistent manner.
  - These left/right comparisons should be made for each individual flight, between successive flights, and cumulated over all flights.

After the census, all flight and observer data must be validated and analysed, as follows:

- Flight Data: detailed analysis of key survey parameters including ground speed, height above ground level, cross track error and time of day.
- Observer Data: detailed analysis of each pair of observers against each other in terms of species identified, numbers counted and group sizes.
- Census Parameter Analysis: analyse observer performance (species identification, numbers seen, group size) against key survey parameters, including height above ground level, ground speed, time of day, up-sun/down-sun effects and type 1, 2 and 3 fatigue (1 = length of sample unit, 2 = hours flown in a day, 3 = total days flown in the census).
- Comparison between Aircraft: comparable analyses of flight data, observer data and census parameter analysis between all aircraft.

We recognise that daily validation is additional work on the survey coordinator; we encourage country survey team to budget for this additional help.

2.7 Post-Census Data Validation

Post census, all flight and observer data will be formally validated – country survey teams are encouraged to do this themselves (and analysis protocols will be made available), though it may

be left to the TAT in the event field resources are insufficient to complete the task on time. The objectives here are threefold:

- First, to detect any residual problems or biases in the data set and rectify them if considered necessary;
- Second, to create the metadata for the census so it is clear to other operators exactly how the census was carried out; and
- Third, to provide feedback for the better design and implementation of future censuses.

At a minimum a summary of the daily validation should be provided at the reporting stage.

Refer to the tables in the overview.

2.7.1 Flight Parameters

A formal analysis is required of each pilot's performance in terms of ground speed, cross-track error and height above ground level, both for individual pilots and for the group of pilots. Hopefully any consistent differences in performance will have been identified and rectified in the field. This analysis will, however, be of great value in the census metadata.

2.7.2 Observer Consistency

A formal analysis is required of the consistency between pairs of observers in terms of the number of sightings and group size of each species. Consistent difference between observers should have been identified and rectified in the field. Nonetheless, this formal analysis will contribute in an important way to the census metadata.

2.7.3 Census Parameters

There are a wide range of census parameters that can affect observer performance and formal analyses should be made. The objective here is to detect any residual problems or biases in the data set, both to evaluate and rectify the data set itself and as feedback into future census design.

An analysis of observer performance, in terms of the number of sightings and the numbers counted will be made against the following important parameters:

- Height agl, strip width and ground speed:
  Experience shows that fewer sightings are made the higher the aircraft, the wider the counting strip and the faster the ground speed. Residual effects of these parameters should be identified and rectified if necessary.
- Time of day
  The visibility of different wildlife can vary significantly according to the time of day. This should be tested for and rectified if there is a large problem.
- Direction of sight
  The direction of sight can be important, especially on North-South flight lines when observers can be looking up-sun or down-sun.

All these analyses should be fully reported as they contribute to the formal census metadata, which will allow others to see exactly how the census was carried out and to compare new censuses against previous ones.

2.8 Data Analysis

Minimum census outputs, including but not limited to:

- Population totals with standard error and confidence limits, by administrative strata, including nominated subdivisions within larger admin areas;
- Distribution maps as grid-level absolute density per 25 km^2, kernel density or graduated symbols;
- Historical data on elephant estimates, and a review of the significance of the current estimate compared to previous estimates;
- Differences and comparison between observers. For each of the more common species, the total numbers of individuals and groups counted by each observer in all transects must be determined. For each observer and each species, the numbers of individual animals and groups that the observer was expected to see should be calculated. For each species, the observed and expected numbers of animals/groups seen should be compared using Chi-square (X2) one sample statistical tests with 1 degree of freedom. Significant differences reported at P<.05;
- Strip width calibration - data for all passes over the airstrip for both observers;
- Differences between observers and photo verified observations (where photos are used);
- Elephant carcasses. Following the method developed by Douglas-Hamilton & Burrill (1991), and adapted by Dunham et al. (2009), the elephant carcass ‘ratio’ (which is a percentage), defined as the ratio of dead elephant (of all categories) to all elephants (dead plus live animals), must be calculated.

    The category 1+2 carcass ratio is defined as the estimated number of elephant carcasses in age category 1 or 2, expressed as a percentage of the sum of this number and the estimated number of live elephants. The 1+2 carcass ratio is an index of the elephant mortality rate during the year of the survey.

- Search Effort. The greater the time spent searching each square kilometre of a transect, the greater the probability that the observer saw animals that occurred within the counting strip. Search effort (in minutes per sq km) for a stratum must be defined as the total time spent flying all transects within that stratum, divided by the total area of those same transects (Gasaway et al. 1986).

2.9 Reporting

The reporting procedures will conform to the below format (Craig 2012).

2.9.1 Narrative Report

The final technical report content shall include the following:

- **Background**
  - location, dates, area description;
  - previous information (eg past surveys);
  - objective;
  - design, stratification, sampling; and
  - power of design.

- **Results**

Tables of results for strata and combined results for strata, separately for:

- Elephants;
- elephants in family groups;
- elephants in bull groups;
- carcass 1;
- carcass 2;
- carcass 3;
- carcass 4;
- carcass ratios, and
- all other species (wild and domesticated).

Each species table should report, for each stratum:

- estimated number of animals;
- number of animals seen in the sample;
- additional animals seen;
- variance of estimate;
- 95% confidence interval;
- PRP (Percentage Relative Precision)/coefficient of variation;
- density;
- maps for each species/attribute with stratum boundaries and showing sighting; and
- positions.

There should be a narrative with any other notable results, eg remarks on carcasses seen.

- **Discussion**

At least the following should be included:

- difference in numbers and precision compared with previous surveys;
- implications of changes in numbers;
- implications of carcasses seen; and
- comments and problems encountered.

- **Literature**
  - sources for previous information on the survey area should be quoted; and
  - sources of methodology/design unique to the survey should be quoted.

- **Appendices**
  - details of methods;
  - crew details; and
  - sampling information: strata, sampling design, areas, and the actual sampling intensities for the stratum (as opposed to the planned sampling intensities).
  - flight information:
    - dates and times, sampling flying, positioning and commuting flying;
    - map of strata and sampling units;
    - map of strata with tracks actually flown;
    - calibration data, including variance estimate;
    - mean ground speed and search rate for each stratum; and
    - mean height flown for the survey and its standard deviation;
  - a comparison of left and right observers; and
  - description of file names and formats for digital data submitted.

2.9.2 Datasets

- **Originals**

Original data sheets should be submitted without transcription.
Original calibration data should also be submitted.

- **Digital copies of data**

The following information should be submitted electronically, per instructions provided by the TAT using the attached table [Attachment 1]:

  - Stratum boundaries (GIS shp. vector files);
  - Geo-referenced landuse and landcover files(GIS shapefiles, decimal degrees);
  - Track logs (actual records of tracks flown, in GIS format);
  - List of strata with: names, areas and sampling intensities;
  - List of species/observations giving alphabetic code (used as identifier in digital records of sightings), numeric code and description; and
  - Sampling unit descriptions consisting of 1 record for each unit with the following fields:
    - name;
    - number;
    - longitude/latitude of start;
    - longitude/latitude of end;
    - width of strip;
    - time of start; and
    - time of end;
  - Description of each sighting consisting of one record for each sighting with the following fields:
    - stratum;
    - sampling unit;
    - species alphabetic code;
    - species numeric code;

        - number seen;
        - in/out;
        - left/right;
        - longitude (decimal degrees); and
        - latitude (decimal degrees).
    - If photographs have been taken, these should be included with, for each, details of the location (GPS) and count data (both photo and visual);
    - Maps of all subunits and blocks (if used);
    - The GIS files defining each stratum and sampling unit; and
    - A comparison of left and right observers.

In the event other wildlife is surveyed, the same procedures are expected.

3 References

1. Craig, G.C. 2012. Monitoring the Illegal Killing of Elephants: Aerial Survey Standards for the MIKE Programme. Version 2.0. CITES MIKE programme, Nairobi.
2. Douglas-Hamilton, I and Burrill, A.1991. Using elephant carcass ratios to determine population trends. In: African wildlife: Research and Management. Eds Kayanja, F.I.B & Edroma, E.L. pp. 98-105. International Council of Scientific Unions, Paris.
3. Dunham, K.M., E. van der Westhuizen, H.F. van der Westhuizen and E. Gandiwa. (2009) Aerial Survey of Elephants and other Large Herbivores in Gonarezhou National Park (Zimbabwe), Zinave National Park (Mozambique) and surrounds. Frankfurt Zoological Society.
4. Norton-Griffiths, M and Frederick, H (2013) “Discussion Paper for Aerial Survey Standards for the Proposed Aerial Census of Elephants in the Selous Game Reserve” TAWIRI, Arusha.
5. Frederick, H., Moyer, D. and Plumptre, A.J. (2010) Aerial Procedures Manual, version 1.0. Wildlife Conservation Society.
6. Gasaway, William C., Stephen D. DuBois, Daniel J. Reed, and Samuel J. Harbo. Estimating Moose Population Parameters from Aerial Surveys. University of Alaska. Institute of Arctic Biology, 1986.

All these references are available through the AfESG Zotero library; http://www.zotero.org/groups/ael
