# Supplementary material for: Continent-wide survey reveals massive decline in African savannah elephants
Source: PeerJ. 2016 Aug 31;4:e2354. doi: 10.7717/peerj.2354 (PMC5012305; doi:10.7717/peerj.2354)

**Methods**

We used simulations to test the accuracy and precision of the two-stage Monte Carlo (MC) procedure that we used to analyze historical trends on GEC sites. We simulated historical data and then used the MC procedure to estimate historical population sizes. We then compared the estimated population trajectories with the “true” (simulated) values.

We ran two sets of simulations, one simulating an individual GEC country with 4 strata (GEC countries had a mean of 3.7 strata with historical data) and one simulating the entire GEC dataset, with 55 strata. Each set of 4 or 55 strata was considered one “replicate,” and we ran 200 replicates for each sample size.

The population in each stratum followed one of four population trajectories over a 20-year period: 1) exponential increase with r = 0.06, 2) exponential decrease with r = -0.06, 3) flat trajectory with r = 0, and 4) symmetric, unimodal trajectory according to the equation $N_{t}=N_{max}e^{-\frac{(t-10.5)}{162}}$, where *t* = years from 1 to 20. For trajectories 1, 2, and 3, initial populations were drawn from a discrete *U*{1, 10000} distribution. For trajectory 4, *N_max_* was drawn from the same distribution. The trajectories of individual strata were determined randomly, with each of the four trajectories having probability = 0.25 of being selected. These four trajectories roughly match the types of elephant population trajectories recorded on GEC sites, which included increasing, decreasing, flat, and unimodal trends.

In the simulations, we treated all historical estimates as sample counts. In reality, 40% of population estimates in the historical dataset were total counts. Thus, confidence limits on simulation results should be somewhat conservative. To estimate the variance of simulated population estimates, we took advantage of the fact that population variance is expected follow a Taylor power law so that log(variance) = α + β·log(population size), where α and β are intercept and slope parameters. We used linear regression to fit a function of this form to the historical and GEC data for elephants (model r^2^ = 0.87) and used the resulting coefficients to predict the variance of the simulated population estimates.

Because historical data were generally sparse ($\bar{x}$ = 5.9 data points per stratum), data for each simulated stratum were limited to year 20, simulating the GEC, as well as 5 randomly selected data points from years 1-19. Time points for individual strata were selected independently so that survey points did not necessarily coincide across strata, as was true for the actual data. For some of replicates at the country level (with 4 strata), no data points were selected for year 1, 1-2, or 1-3 by chance. As a result, for these replicates, inference was restricted to years with data available. When results for all 200 replicates were compiled, this resulted in slightly smaller sample size for year 1-3 than for other years but should have no effect on our conclusions.

For each of the 200 replicates, we used the MC procedure described in the Methods to estimate historical populations for all strata combined. We made inferences based on the mean of the resulting 1,000 MC estimates, as our goal was to determine the accuracy and precision of mean model predictions. For each replicate and year, we measured the percentage bias in MC estimates by taking the absolute value of the difference between the mean MC estimate and the “true” population size and dividing the difference by the true population size.

**Results and discussion**

For the single-country scenario with four simulated strata, MC estimates were nearly unbiased, differing by a mean of 0.7% from “true” population sizes (range of bias values by year: 0.4-2.4%; Article S1 Fig. 1). Confidence limits on percentage differences between MC estimates and true population sizes were wide for the earliest years in the trajectory but narrowed considerably thereafter. For years 11-20, confidence limits on MC predictions were within ±8% of true estimates. Thus, MC estimates for early years at the country level are imprecise, but MC estimates for the second half of the time series should be more precise.

For the GEC-wide scenario with 55 simulated strata, MC estimates also showed little bias, differing by a mean of 0.9% from “true” population sizes (range of bias values by year: 0.6-1.2%; Article S2 Fig. 2). Confidence limits on percentage differences were much narrower than those for the single-country scenario. With 55 strata, confidence limits for all years were within a mean of ±4% of true values, and confidence limits for years 11-20 were within ±2% of true values.

We conclude that for either sample size, our MC procedure is able to reveal underlying population trends with little bias. For sample sizes equivalent to a single GEC country, estimates for early years in the time series may be imprecise, but estimates for the second half of the time series were far more precise. For the entire GEC dataset, MC estimates in all years should be within a few percent of true values.

Article S1 Figure 1. Results from 200 simulations of the MC method used to estimate historical trends, with 4 simulated strata. Solid line indicates the mean of 200 percentage differences between MC estimates and “true” population sizes. Shaded area indicates 95% confidence limits for the percentage difference. For reference, dashed line indicates 0% difference between MC estimates and true population size. Note that estimates of bias in the text were based on the absolute value of the percentage difference graphed here.


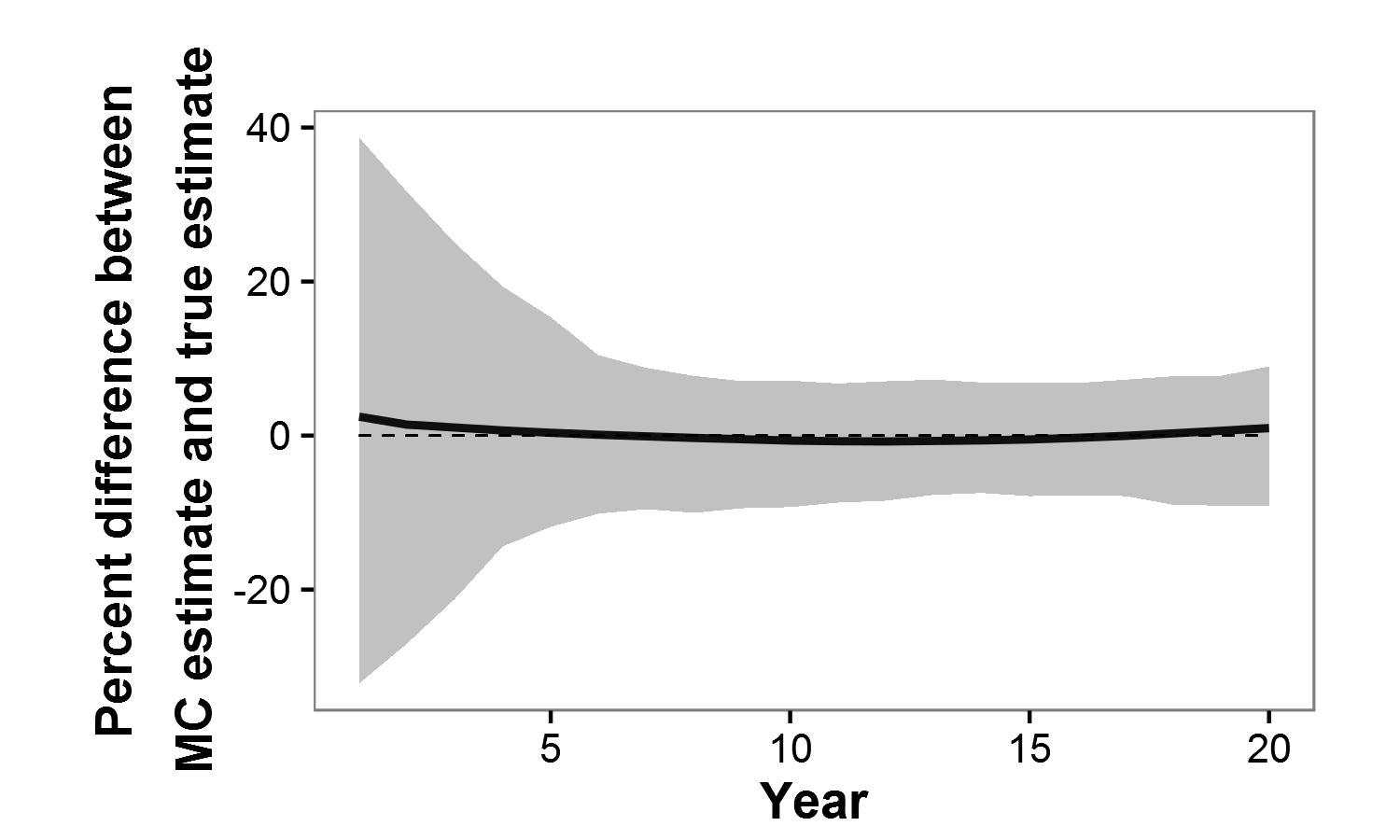


Article S1 Figure 2. Results from 200 simulations of the MC method used to estimate historical trends, with 55 simulated strata. Solid line indicates the mean of 200 percentage differences between MC estimates and “true” population sizes. Shaded area indicates 95% confidence limits for the percentage difference. For reference, dashed line indicates 0% difference between MC estimates and true population size. Note that estimates of bias in the text were based on the absolute value of the percentage difference graphed here.


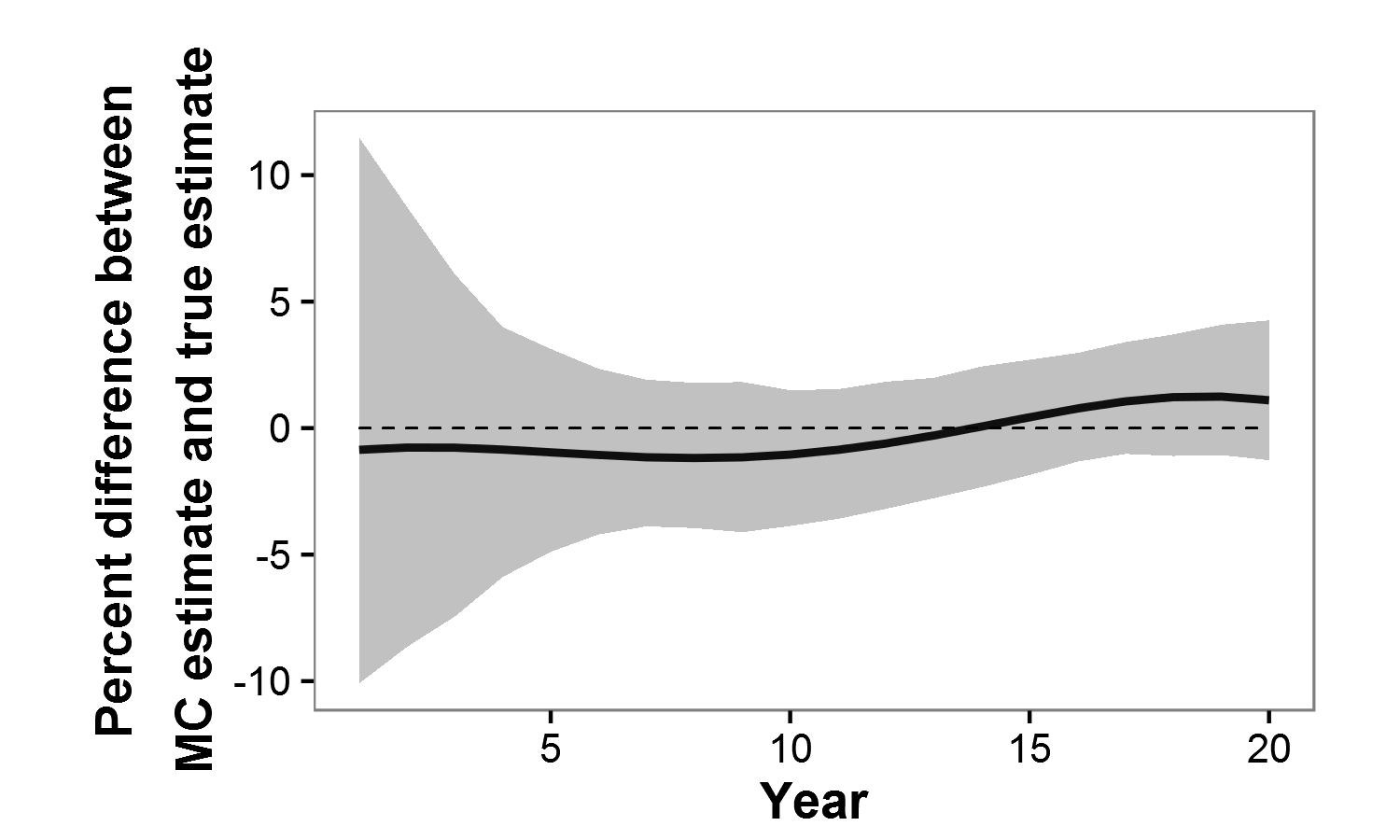

Supplement: Article S2 [file peerj-04-2354-s014.docx]
